# The Network Zoo: a multilingual package for the inference and analysis of gene regulatory networks

**DOI:** 10.1186/s13059-023-02877-1

**Published:** 2023-03-09

**Authors:** Marouen Ben Guebila, Tian Wang, Camila M. Lopes-Ramos, Viola Fanfani, Des Weighill, Rebekka Burkholz, Daniel Schlauch, Joseph N. Paulson, Michael Altenbuchinger, Katherine H. Shutta, Abhijeet R. Sonawane, James Lim, Genis Calderer, David G.P. van IJzendoorn, Daniel Morgan, Alessandro Marin, Cho-Yi Chen, Qi Song, Enakshi Saha, Dawn L. DeMeo, Megha Padi, John Platig, Marieke L. Kuijjer, Kimberly Glass, John Quackenbush

**Affiliations:** 1 Department of Biostatistics, Harvard T.H. Chan School of Public Health, Boston, MA USA; 2 Present Address: Biology Department, Boston College, Chestnut Hill, MA USA; 3 Channing Division of Network Medicine, Brigham and Women’s Hospital and Harvard Medical School, Boston, MA USA; 4 Present Address: Lineberger Comprehensive Cancer Center, University of North Carolina at Chapel Hill, Chapel Hill, NC USA; 5 Present Address: CISPA Helmholtz Center for Information Security, Saarbrücken, Germany; 6 Present Address: Genospace, LLC, Boston, MA USA; 7 Department of Biochemistry and Molecular Biology, Pennsylvania State University College of Medicine, Hershey, PA USA; 8 Present Address: Department of Medical Bioinformatics, University Medical Center Göttingen, Göttingen, Germany; 9 Present Address: Center for Interdisciplinary Cardiovascular Sciences, Division of Cardiovascular Medicine, Department of Medicine, Brigham and Women’s Hospital, Boston, MA USA; 10 Department of Molecular and Cellular Biology, University of Arizona, Tucson, AZ USA; 11 Present Address: Monoceros Biosystems, LLC, San Diego, CA USA; 12 Center for Molecular Medicine Norway, Nordic EMBL Partnership, University of Oslo, Oslo, Norway; 13 Department of Pathology, Leiden University Medical Center, Leiden, The Netherlands; 14 Present Address: Department of Pathology, Stanford University School of Medicine, Palo Alto, CA USA; 15 Present Address: School of Biomedical Sciences, Hong Kong University, Pokfulam, Hong Kong; 16 Expert Analytics AS, Oslo, Norway; 17 Dana-Farber Cancer Institute, Boston, MA USA; 18 Present Address: Institute of Biomedical Informatics, National Yang Ming Chiao Tung University, Taipei, 112 Taiwan; 19 Present Address: Computational Biology Department, Carnegie Mellon University, Pittsburgh, PA USA; 20 Leiden Center for Computational Oncology, Leiden University, Leiden, The Netherlands

**Keywords:** Gene regulation, Multi-omic analysis, Network biology, Open-source software

## Abstract

Inference and analysis of gene regulatory networks (GRNs) require software that integrates multi-omic data from various sources. The Network Zoo (netZoo; netzoo.github.io) is a collection of open-source methods to infer GRNs, conduct differential network analyses, estimate community structure, and explore the transitions between biological states. The netZoo builds on our ongoing development of network methods, harmonizing the implementations in various computing languages and between methods to allow better integration of these tools into analytical pipelines. We demonstrate the utility using multi-omic data from the Cancer Cell Line Encyclopedia. We will continue to expand the netZoo to incorporate additional methods.

**Supplementary Information:**

The online version contains supplementary material available at 10.1186/s13059-023-02877-1.

## Background

Biological phenotypes are driven by a complex network of interacting elements that defines cell types and determines response to perturbations [1]. These interactions can be modeled by assessing the physical binding between biological elements [2], their co-expression [3], and their co-dependency [4] to identify functional modules that together control the emergence of a given phenotype. A particular type of network is gene regulatory networks (GRNs) that are comprised of regulators and their target genes. One type of regulators is transcription factors (TFs), regulatory proteins that bind to DNA to activate or repress gene transcription. TFs often form complexes that act together to regulate transcription [5–7] and TF activity can be further influenced by epigenetic modifications such as promoter methylation or histone acetylation [8]. Other regulators of gene expression include microRNAs (miRNAs) that act post-transcriptionally, primarily to degrade and subsequently repress the expression of their mRNA target [9, 10]. These and other factors together modulate the expression of the more than twenty-five thousand protein-coding genes in the genome, altering cellular processes and giving cells the potential to respond to various stimuli [7].

Despite rapid advances in sequencing technologies, the size and complexity of GRNs put them out of reach of direct measurement [6]. Consequently, there have been many attempts to represent them using computational methods [3, 6, 11–13], although not all model gene regulatory processes.

Our group has developed a number of robust methods for GRN inference and analysis (Additional file 1: Text S1), each of which takes advantage of multiple data types available in individual studies. Each method is based on using known biological interactions as prior information to guide network inference from the data, seeking consistency between a variety of input data sources to identify a common underlying biological signal. Our methods for reconstructing networks include PANDA [14] that infers a cis-regulatory network for TFs and their target genes by first positing a prior regulatory network and then iteratively optimizing its structure by seeking consistency between gene co-expression and TF protein-protein interactions (PPIs). The prior regulatory network can be constructed by scanning the sequence of the promoter region of target genes (for example, by using FIMO [15]) for transcription factor binding sites (TFBS) using TF motifs taken from catalogs (such as CIS-BP [5]). The input TF PPI data can be obtained from resources such as STRING [2], and gene co-expression is obtained from the particular experiment being analyzed. The inference is based on the concept that interacting TFs co-regulate their target genes and co-expressed genes are potentially regulated by the same sets of TFs. PANDA uses message passing to iteratively update all three data sets, maximizing consistency between them, until it converges on a data set-specific regulatory network with interaction scores between TFs and their regulated targets. OTTER [16] takes the same input but uses graph matching as an alternative implementation of the network optimization solution. SPIDER [17] uses epigenetic data such as DNase-Seq measurements of DNA accessibility to inform the PANDA prior network on context-specific accessible chromatin regions. EGRET [18] uses cis-eQTL data to seed the method with genotype-specific priors. PUMA [19] extends PANDA’s regulatory framework by including miRNA target predictions in the initial prior network to capture both TF and miRNA regulation of target genes/mRNAs.

LIONESS [20] is a general-purpose single-sample network method that can be used with any network inference approach. It iteratively leaves out individual samples and uses linear interpolation to infer sample-specific networks for each sample in the original sample set. LIONESS outputs individual sample edge weights which can be treated as inferred measures on each sample, allowing statistical comparisons to be performed on the associated networks. A key use case of LIONESS is to infer sample-specific GRNs using PANDA. DRAGON [21] is a flexible method for integrating multiple data sources into a Gaussian Graphical Model (GGM). GGMs differ from correlation networks in that partial correlation corrects for spurious correlations between variables; the multi-omic networks inferred by DRAGON therefore represent direct associations between the different data types included for network inference. DRAGON differs from PANDA and similar methods for network inference in that GGMs are undirected unipartite networks rather than a bipartite GRNs.

A second group of methods in netZoo was developed to identify and explore higher-order structure in GRNs [22, 23] by identifying highly connected network “communities” and comparing the structure of these communities between phenotypic states. CONDOR [24] identifies communities in bipartite graphs [25] (including eQTL and TF-gene networks), while ALPACA [26] finds differential community structures between two networks, such as in a case versus control setting, by going beyond the simple difference of edge weights and using the complete network structure to find differential communities. CRANE [27] assesses the significance of differential modules discovered by ALPACA based on a baseline of network ensembles that are simulated while preserving the specific structure and constraints of GRNs. In this regard, CRANE provides an efficient tool for hypothesis testing inference on differential community structures in GRNs. A fourth method, MONSTER [28], treats the transition between related biological states as one in which a first network is subject to a regulatory transition that involves altering transcription factor connections to their target genes. Mathematically, MONSTER estimates such changes by identifying a “transition matrix” that maps an initial state network to a final state network to identify the TFs that have the largest effect on the structure of the network and therefore are likely to help drive the phenotypic transition. SAMBAR [29] allows users to group biological samples based on how genetic variants alter functional pathways, and finally, YARN [30] is a tissue-aware implementation of smooth quantile normalization for multi-tissue gene expression data.

Many of these methods share a methodological and philosophical framework that derives from the “No Free Lunch Theorem”—modeling of complex systems can be improved by incorporating domain-specific knowledge [31] — as they optimize around a regulatory network prior and impose biologically motivated soft constraints. Many of these methods also use an overlapping set of standard input data types and provide complementary views of GRNs. As such, they have often been used together. To facilitate their use and integration into analytical pipelines, we gathered these into the Network Zoo (netZoo; netzoo.github.io), a platform that harmonizes the codebase for these methods, in line with recent similar efforts [32, 33], and provides implementations in R, Python, MATLAB, and C. In building netZoo, we also created the ZooKeeper, an online server that helps ensure consistency of the codebase as it is continuously updated in response to user feedback. The netZoo codebase has helped develop an ecosystem of online resources for GRN inference and analysis to both scientists and method developers that includes tools to integrate contributions from the community, to share use cases [34], and to host and visualize networks online [35].

To demonstrate the power of this unified platform, we used netZoo methods to build a comprehensive collection of genome-scale GRNs for the cell lines in the Cancer Cell Line Encyclopedia (CCLE) [36–38]. We also used PANDA, LIONESS, and MONSTER to infer TF-gene targeting in melanoma to explore how regulatory changes affect disease phenotype, and used DRAGON to integrate nine types of genomic information and find multi-omic markers that are associated with drug sensitivity.

## Results

### The netZoo integrates network inference and downstream analyses

Regulatory processes drive gene expression and help define both phenotype and the ability of a biological system to respond to perturbations. However, identifying context-specific regulatory processes is difficult because the underlying regulatory network is often unobserved [6]. Several netZoo methods address this challenge by integrating multiple sources of available data to infer TF-gene regulation.

**Fig. 1:**
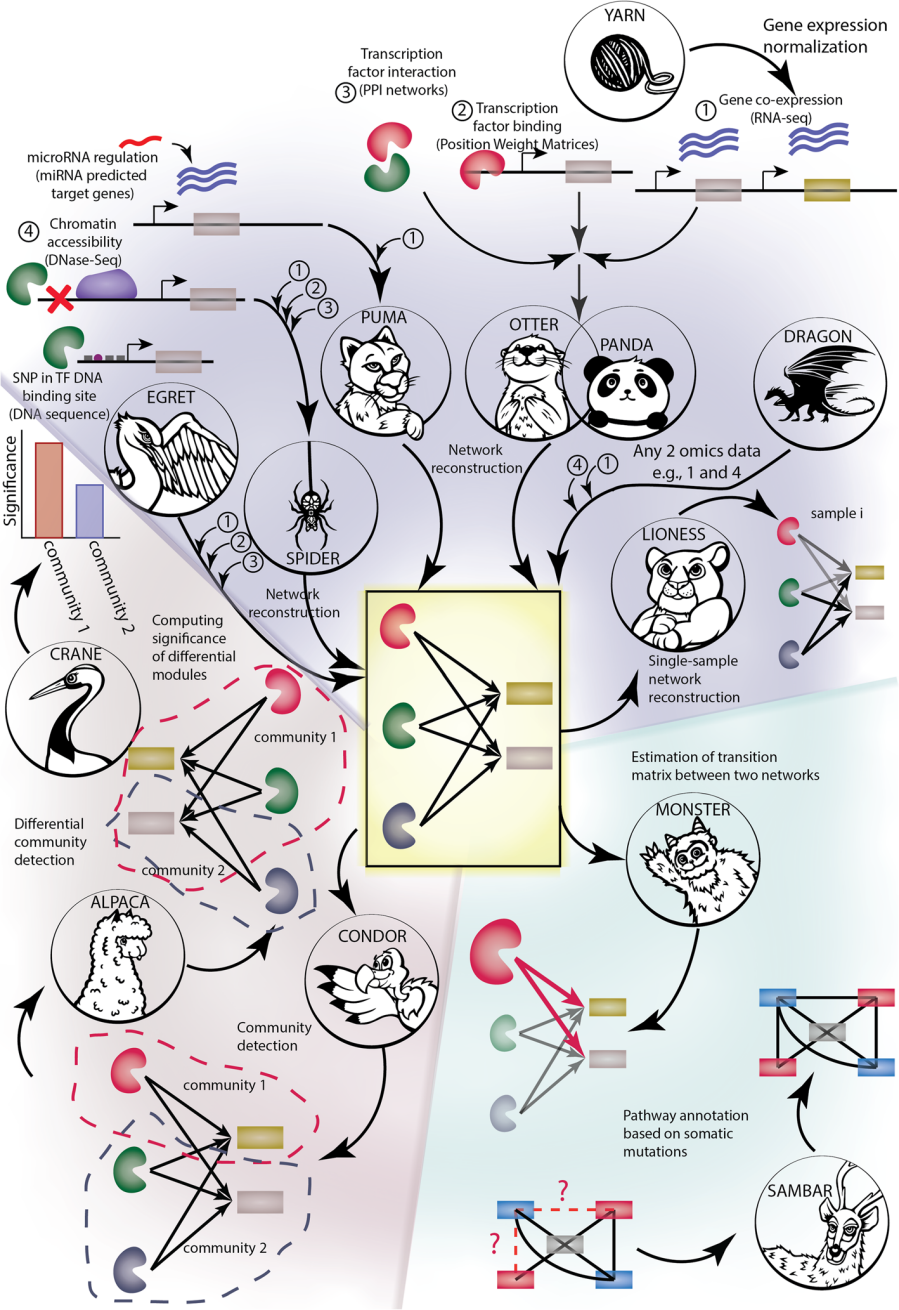
Inference and analysis of GRNs using netZoo. YARN normalizes gene expression (RNA-Seq) data to account for differences between tissues. Then, a first group of methods uses normalized gene expression data to infer gene regulatory networks (PANDA, PUMA, OTTER, LIONESS, SPIDER, EGRET) to reconstruct GRNs using multiple genomic modalities. The input data used for PANDA and OTTER are normalized RNA-Seq data to build gene coexpression networks, PPI network such as STRINGdb to build TF interaction networks, and a prior knowledge TF motif network built on scanning TF position weight matrices in promoter region of target genes. We refer to these three input networks as the core input data that may be shared by groups of methods. In addition to this core input, SPIDER uses DNase-Seq chromatin accessibility data to constrain predictions to open regions of the genome. Instead of using TF motif network, PUMA employs miRNA target gene prediction data from tools such as TargetScan and miRanda as a prior knowledge network to seed inference of miRNA regulation networks. EGRET uses data from DNA sequence to first identify variants in TF binding sites and compute their effect on target gene regulation by combining these mutation data with the core input data. DRAGON builds multi-omic, partial correlation-based networks that can use data such as RNA-seq, methylation status, protein levels, and chromatin accessibility. A second group (CONDOR, ALPACA, CRANE) identifies communities in the networks (CONDOR), finds differential community structures between two networks of interest (ALPACA), and estimates the significance of differences between modules (CRANE). Finally, MONSTER estimates a transition matrix between two networks representing an initial and a final state, and SAMBAR de-sparsifies mutation data using biological pathways. Overlapping methods share the same input data. SNP, single nucleotide polymorphism; PPI, protein-protein Interaction network; miRNA, microRNA

Many of the netZoo methods share common methodological and computational cores, and over the years, we have used combinations of these methods to explore the regulatory features driving biological states [39, 40]. Harmonizing the implementation of these methods to create netZoo as a unified resource facilitates interoperability and their seamless integration in a pipeline that connects network inference with downstream analyses (Fig. 1; Table S1) to generate hypotheses and actionable biological insights. To do so, we aggregated methods in a unique central resource, which allowed to reconcile their dependencies and standardize the formats of input data and the output of generated networks. This facilitated building interfaces between them by identifying intersection points in algorithms and by using data transformation to fit underlying statistical hypotheses for each method. Co-developing methods in various languages while using the same unit tests across them has helped identify inconsistencies for some edge cases and has dramatically improved reproducibility. netZoo implementations were also optimized for runtime and memory usage which included using GPU [41], and wrapping faster implementations to be used in other languages.

To demonstrate these features, we chose to model gene regulation in CCLE cell lines that include measurements for various omics but not for the activity of regulatory elements, which further supports the need for GRN inference. CCLE data presents unique challenges first to identify meaningful associations in multi-omic data with different underlying distributions and second to infer single-sample networks using one gene expression measurement per cell which may occur after collapsing replicates. netZoo is uniquely positioned for GRN inference in this setting because of the connections we created between existing methods, which enable analyses that were not previously possible. The package also includes novel methods that were designed for large-scale multi-omic data such as CCLE. Finally, netZoo implementations are optimized for runtime and memory and can scale up network inference for various omics, cell lines, and node types such as thousands of genes and their targeting regulatory elements.

### Estimating TF targeting in melanoma CCLE cell lines

Melanoma progression and metastasis are known to be associated with many regulatory changes that alter patterns of gene expression [42], ultimately leading to phenotype switching to malignancy and drug resistance. These changes in expression can be tied to a variety of regulatory elements including transcription factor targeting, miRNA suppression of transcripts, and genomic and epigenetic changes. To demonstrate the utility of combining netZoo methods, we applied PANDA with LIONESS to model transcriptional regulation for individual samples in melanoma. This workflow allows us to understand regulatory changes in disease by inference and analysis of sample-specific regulatory networks for the 76 melanoma cell lines available in CCLE and exploring a variety of disease-associated processes (see the sections “Methods”: “Applications of netZoo using the Cancer Cell Line Encyclopedia”).

First, we used PANDA to generate an aggregate network across all CCLE cell lines, and we derived single-sample networks using LIONESS (see the sections “Methods”: “Applications of netZoo using the Cancer Cell Line Encyclopedia”). Then, we used ANOVA to analyze the 76 melanoma cell line networks to explore whether TF targeting scores, the sum of outgoing edge weights for each TF in the network [43], could be linked to methylation changes and copy number alterations (see the sections “Methods”: “TF targeting analysis”).

**Fig. 2:**
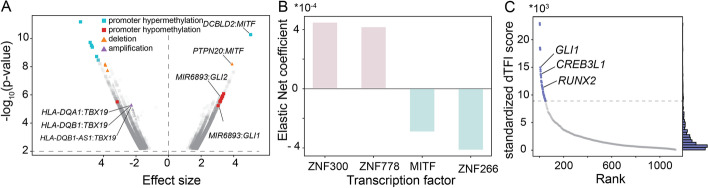
Modeling regulatory processes in melanoma using CCLE data. **A** Volcano plot of the ANOVA associations between TF targeting scores and promoter methylation and copy number statuses in melanoma cell lines. The 10 largest significant associations are colored in red and cyan for methylation status, and in orange and purple for copy number status. **B** Elastic net regression of regorafenib cell viability on TF targeting scores in melanoma cell lines. The figure represents the two largest positive coefficients and two largest negative coefficients. **C** Differential TF involvement in the transition between primary melanoma cell line and a cell line derived from melanoma metastasis. The top 50 TFs are colored in blue

Among the top ten associations (Fig. 2A), we found that targeting by melanocyte-inducing transcription factor (MITF) was associated with changes in promoter methylation; in particular, we found a significant association between MITF targeting score (see the sections “Methods”: “TF targeting analysis”) and promoter hypermethylation of Discoidin, CUB, and LCCL Domain Containing 2 (*DCBLD2*) (Fig. 2A). We also found that MITF targeting was associated with the deletion of Protein Tyrosine Phosphatase Non-Receptor Type 20 (*PTPN20*; Fig. 2A). The targeting by TFs (see the sections “Methods”: “Applications of netZoo using the Cancer Cell Line Encyclopedia”) glioma-associated oncogenes 1 and 2 (GLI1 and GLI2) was also significantly increased in melanoma. In examining GLI1 and GLI2 targeting, we found it to be associated with promoter hypomethylation of *MIR6893*. Finally, mining additional significant associations (Fig. 2A), we find a decrease of targeting by TBX19 to be associated with the amplification of the *HLA-DBA1* and *HLA-DQB1* genes.

We also tested whether TF targeting in the CCLE melanoma cell lines was associated with response to regorafenib, a multi-kinase inhibitor that has been approved for treating metastatic colorectal cancer, advanced gastrointestinal stromal tumors, and advanced hepatocellular carcinoma. The drug has been shown to have a high affinity for BRAF [44], a kinase commonly mutated in metastatic melanoma, suggesting it may also show efficacy in treating melanoma. We conducted elastic net regression [45] on TF targeting scores to test for meaningful pharmacogenomic interactions [46] associated to cell viability after regorafenib treatment (see the sections “Methods”: “TF targeting analysis”), and among the largest four variables’ importance, we found targeting by MITF to be negatively associated with cell viability, while targeting by ZNF778 was positively associated to it (Fig. 2B, Additional file 1: Fig. S1).

This PANDA-LIONESS combination has been applied previously in various other settings, such as the study of sex differences in health and disease [47], by our group [20, 39, 48] and others [49], but the uniqueness of the CCLE data makes this combination particularly powerful on single gene expression samples from collapsed replicates and even in the absence of replicates. To demonstrate the advantage of using netZoo methods in combination, we modified MONSTER to use LIONESS networks as input for the estimation of drivers of transitions between two biological states using a PANDA-LIONESS-MONSTER pipeline (see the sections “Methods”: “TF targeting analysis”). We did this to study severe forms of melanoma and their transition from a noninvasive to an invasive state [50] which can be driven by epithelial to mesenchymal transition (EMT). We used MONSTER to define a TF transition matrix that maps a nonmetastatic LIONESS network for a cell line derived from a primary tumor (Depmap ID: ACH-000580) to a LIONESS network of a cell line derived from melanoma metastasis (Depmap ID: ACH-001569) (see the sections “Methods”: “TF targeting analysis”). We found that the TFs RUNX2, GLI1, and CREB3L1 were among those with the largest differential involvement score [28] (Fig. 2C), indicating that they have the most profound changes in their regulatory targets as cells become metastatic.

### CCLE pan-cancer analysis reveals meaningful regulatory interactions

The CCLE cell lines are among the most widely studied model systems available in oncology research and include a large number of measurements for various biological entities as well as viability assays following drug challenges and gene knockouts. We used DRAGON [21,51] to explore multi-omic associations captured in these data, taking advantage of covariance shrinkage [51,52] to account for the unique structure of each data type. We calculated DRAGON partial correlation networks between all pairwise sets of measurements on the CCLE cell lines (see the sections “Methods”: “Computing CCLE multi-omic associations”), but we will focus on four sets of partial correlations in our analysis: (1) miRNA levels and gene knockouts, (2) protein levels with metabolite levels, (3) cell viability assays after drug exposure and gene knockout screens, and (4) TF targeting and metabolite levels.

**Fig. 3:**
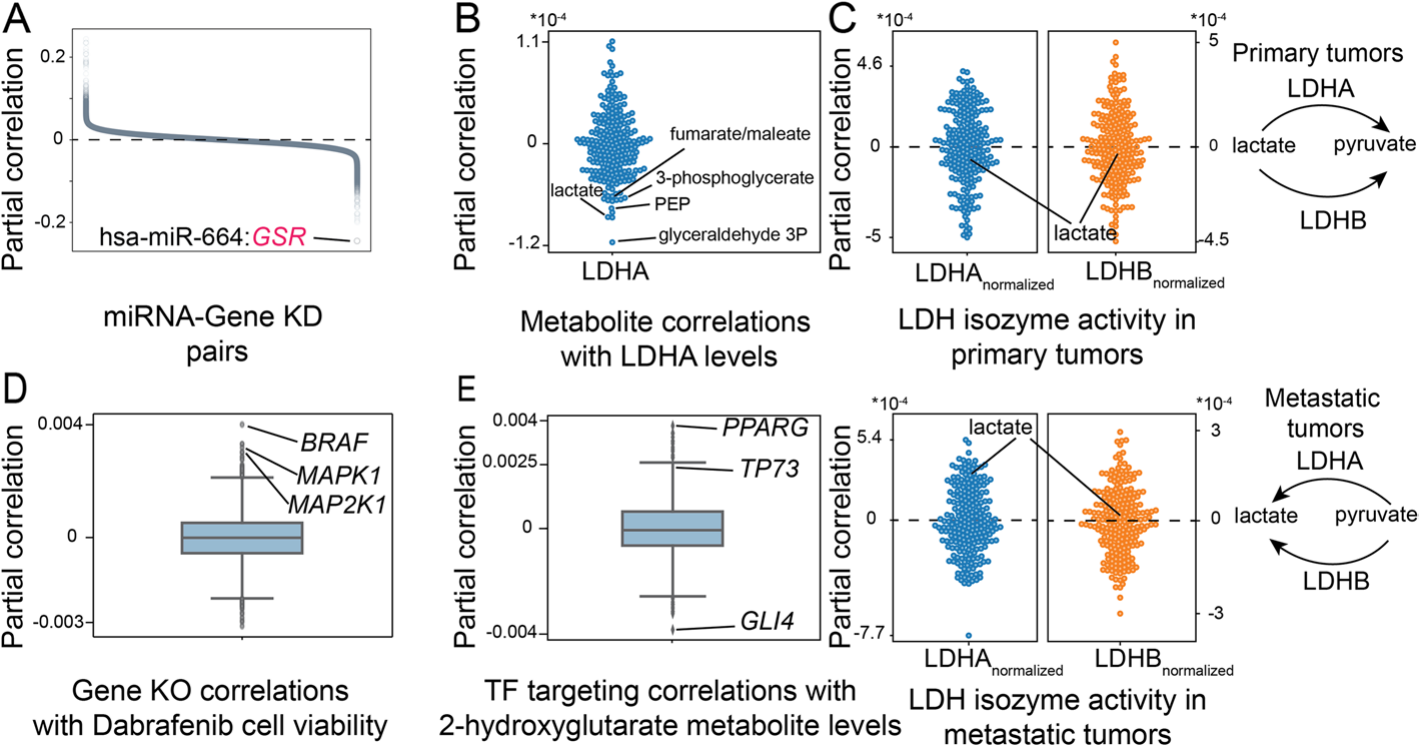
Pan-cancer analysis of regulatory interactions using DRAGON. **A** Partial correlation between miRNA levels and gene knockout screen across all cancer cell lines. **B** Partial correlation of metabolite levels and *LDHA* protein levels. **C** Partial correlation between normalized *LDH* isozyme levels and lactate in cell lines from primary and metastatic tumors. **D** Partial correlation between gene knockout screens and dabrafenib cell viability assays. **E** Partial correlation between 2-hydroxyglutarate levels and TF targeting across all cancer cell lines

In the first comparisons between miRNA expression and gene knockout, we assume that strong gene silencing by miRNA [9] would share a similar dependency signature with a gene knockout using clustered regularly interspaced short palindromic repeats (CRISPR), as other small RNAs are commonly used in knockdown experiments. We found that *MIR664* levels have a strong partial correlation with glutathione-disulfide reductase (*GSR*) dependency (Fig. 3A).

In our second DRAGON analysis of metabolomic and proteomic data, we first found three glycolysis metabolites, phosphoenolpyruvic acid, 3-phosphoglycerate, and glyceraldehyde 3P, had a negative partial correlation with lactate dehydrogenase-A (LDHA) protein levels (Additional file 1: Fig. S2, Fig. 3B), which converts pyruvate to lactate as the last step of glycolysis. This suggests that these metabolites are upstream of LDHA and indicates an active glycolysis (Additional file 1: Fig. S2). Second, we found that fumarate/maleate levels, which are TCA cycle metabolites, were negatively partially correlated with LDHA (Fig. 3B), indicating lower TCA cycle intermediates levels when LDHA is active. To further confirm the production of lactate in cancer cell lines, we correlated the activity of LDHA with lactate concentrations by normalizing it by LDHB levels (see the sections “Methods”: “Computing CCLE multi-omic associations”), the isozyme that preferentially carries the backward reaction to produce pyruvate. We find that normalized LDHA levels and lactate are positively correlated, while normalized LDHB levels and lactate are negatively correlated, which confirms the known preferential directions for these enzymes, reflecting their larger molecular affinities towards lactate and pyruvate. We also find that normalizing by the isozyme ratio is an essential step to account for the activity of LDH depending on the levels of LDHA and LDHB chains, thereby avoiding spurious correlations (Fig. S3). Using these normalized variables, we further investigated metabolic phenotypes in two groups of cells based on their origin, either primary or metastatic. We find that in metastatic tumors, both LDHA and LDHB produce lactate, while in primary tumors, both LDHA and LDHB use lactate as a substrate to produce pyruvate (Fig. 3C).

We also employed DRAGON to analyze cell viability assays after drug exposure and CRISPR screens (see the sections “Methods”: “Computing CCLE multi-omic associations”). Not surprisingly, we found that viability after exposure to dabrafenib, a BRAF inhibitor, was highly correlated with *BRAF* knockout (Fig. 3D). Dabrafenib cell viability was also correlated to *MAPK1* and *MAP2K1*, two genes that are downstream of BRAF in the MAPK signaling pathway.

Finally, motivated by recent findings implicating oncometabolites in altering the epigenetic landscape in cancer [53], we analyzed oncometabolite 2-hydroxglutarate (2HG) levels because it has been shown to induce the hypermethylator phenotype in glioma and acute myeloid leukemia by inhibiting histone demethylases [54]. To identify TFs that are associated with 2HG epigenetic regulation, we computed correlations between TF targeting and 2HG levels across all CCLE cell lines using DRAGON (Fig. 3E). We found that 2HG levels might affect the regulatory profile of several TFs including PPARG, TP73, and GLI4.

### An integrated CCLE multi-omic network portal

Having inferred DRAGON networks for additional pairwise combinations of measurements (Table S2) on the CCLE cell lines, we integrated these partial correlation networks from various biological data types and created an online portal to allow exploration of the integrated relationships we discovered (Additional file 1: Fig. S4; see the sections “Methods”: “CCLE pan-cancer map”). First, promoter methylation status, copy number variation, histone marks, and miRNA partial correlations networks with gene expression were stacked to capture the multi-modal regulation of gene expression. Then, gene expression was linked to protein levels, which in turn was associated with cellular phenotypes represented by metabolite levels, drug sensitivity, and cell fitness resulting in a final genotype-to-phenotype map.

**Fig. 4:**
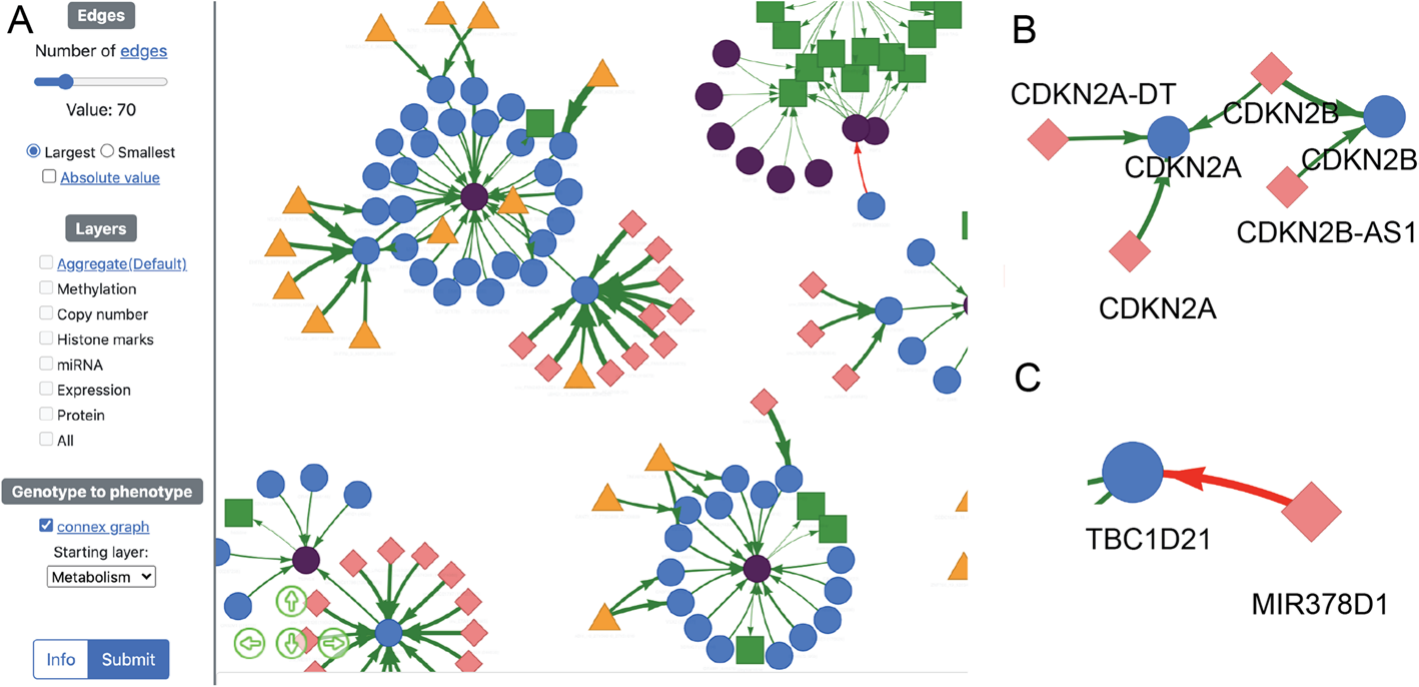
Multi-tiered CCLE map links genotype to cellular phenotypes. **A** Screenshot of the online resource accessible at https://grand.networkmedicine.org/cclemap/ that uses DRAGON to link promoter methylation (orange triangle), copy number variation (pink diamond), histone marks, miRNA levels, gene expression (blue circle), protein levels (purple circle), metabolite levels (green square), drug sensitivity, and cell fitness following CRISPR gene knockout. Green arrows indicate positive partial correlation and red arrows indicate negative partial correlation. **B** Positive partial correlations between copy number variation and gene expression of *CDKN2A* and *CDKN2B*. **C** Negative partial correlation between *MIR378D1* levels and *TBC1D21* expression

The resulting integrated CCLE partial correlation network is available online (https://grand.networkmedicine.org/cclemap/) and can be queried to explore the biological associations contained within (Fig. 4A). To illustrate the utility of this multi-tiered correlation network map, we used it to examine the effect of copy number variation on gene expression. As expected, we found positive partial correlations between copy number and expression. For example, we not only found that *CDKN2A* and *CDKN2B* copy numbers have a positive partial correlation with *CDKN2A* and *CDKN2B* expression, respectively (Fig. 4B), but that *CDKN2B* copy number is correlated with *CDKN2A* expression, which may reflect the fact that these two genes are adjacent in the genome. We also found negative partial correlations between copy number variation and gene expression. For example, *MIR378D1* copy number is negatively partially correlated with *TBC1D21* expression (Fig. 4C), suggesting that *TBC1D21* may be repressed by *MIR378D1*. Although *TBC1D21* is not listed as a target of *MIR378D1* in miRDB, other members of the *TBC1* family, including *TBC1D12* (Target Score (TS) 66), *TBC1D16* (TS 61), and *TBC1D24* (TS 53), are among its predicted targets [55].

### Creating a community ecosystem for collaborative software development

Development of netZoo has been driven through collaborative work involving users and developers at several academic institutions, all of whom are committed to open-source, community-driven method development. A great deal of our work in harmonizing the code has been to facilitate reproducibility across the implementation of related methods, to facilitate re-use of common methods for network inference, and to standardize input and output file formats to enable the creation of network analysis pipelines.

**Fig. 5:**
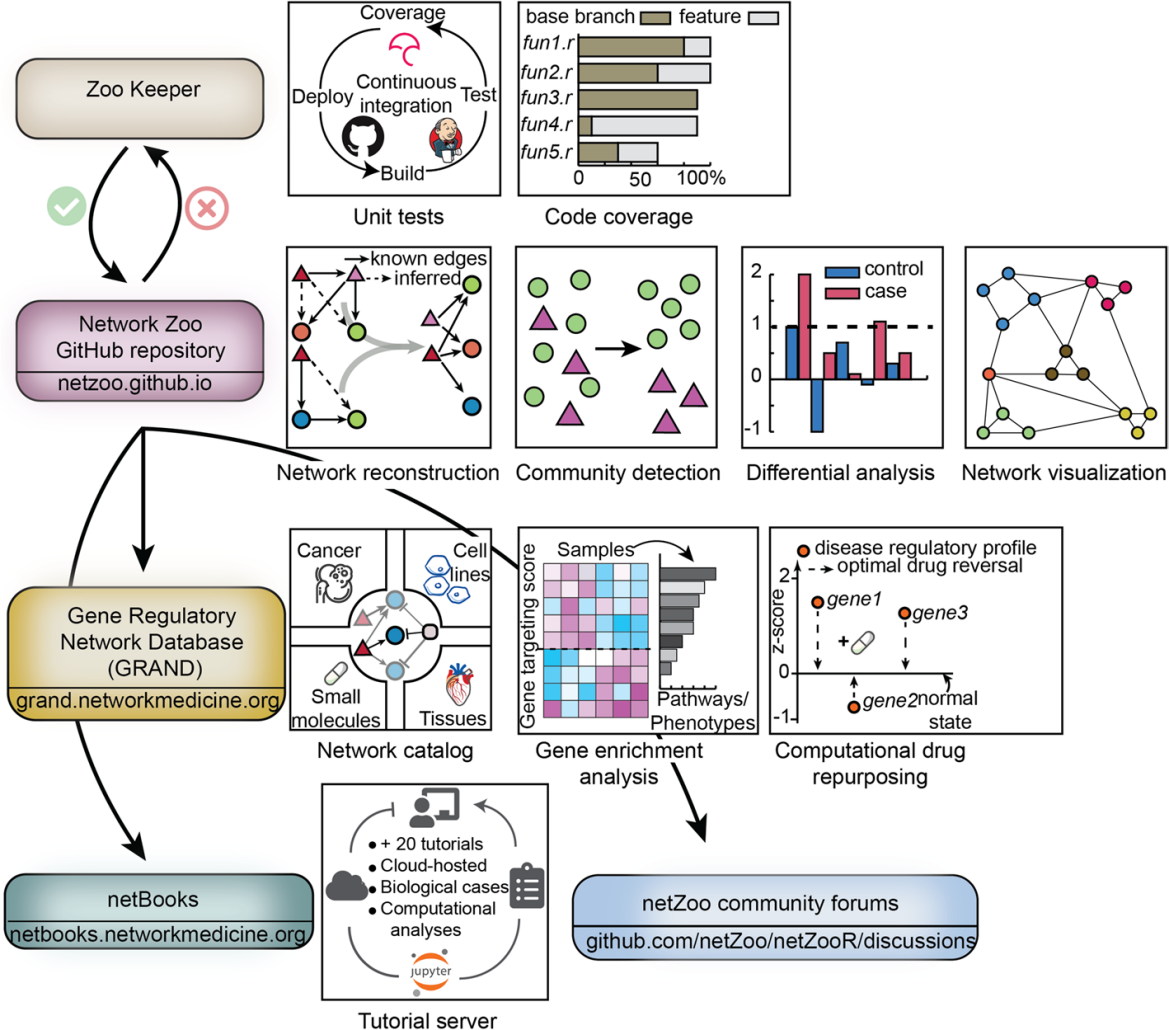
netZoo ecosystem. The codebase is hosted on GitHub and is regularly tested through a continuous integration system called ZooKeeper. Networks generated by netZoo methods are hosted in the GRAND database. Cloud-hosted use cases and tutorials are available through a JupyterHub server called Netbooks. GitHub discussions and issues provide a forum for discussion and exchange within the community.

The netZoo codebase is version-controlled in GitHub and implementations of most methods are available in R [56, 57], Python [58], MATLAB [59], and C (Fig. 5). These implementations were developed over the years for various needs for performance, ease of use, and ease of combination with plotting and downstream statistical analysis functions that each programming language may offer. Using a synchronized resource for code development avoids creating parallel branches and gives users access to tested and optimized methods that are up to date with the newest frameworks, particularly for the growing userbase in R and Python, as well as with third-party dependencies. The codebase includes additional helper functions for plotting and analysis, and GPU-accelerated implementations [41] for faster network inference across large numbers of samples. The netZoo codebase is part of a larger ecosystem of online tools, that together support reproducible science. A first component of this ecosystem is a continuous integration tool ZooKeeper that runs unit tests using both public GitHub actions and a custom server, to regularly test the code and to maintain the integrity of the software and update its dependencies to third-party software. This tool facilitates contributions from the community using a fork-branch model; new contributed features are tested through ZooKeeper before being added to the core codebase. A second component called Netbooks allows access to a set of cloud-based Jupyter notebook use cases and tutorials [34]. Finally, GRAND database can store genome-scale networks and visualize them on the browser [35]. These online tools are essential to conducting large-scale analyses because most public hosting services cannot host genome-scale networks, and public cloud servers often do not offer enough memory to analyze these networks. These tools are constantly updated beyond their initial content, for example, we released Netbooks 2.0 which adds new use cases, as a companion to this work, and we will continue to develop these tools together as new releases of the codebase will enable the generation of additional networks hosted in GRAND and new analyses in Netbooks, together supporting all the aspects of high-quality and reproducible research in computational biology.

## Discussion

We used netZoo methods to identify disrupted regulatory processes in melanoma, then we extend the analysis by building a pan-cancer multi-omic map across all CCLE cell lines. In the cancer type-specific use case, we used netZoo methods to model gene regulation in CCLE melanoma cell lines by first analyzing multi-omic associations between TF targeting and various genomic modalities, then comparing them with drug sensitivity to identify markers of resistance, and finally identifying TFs that drive the transition to metastasis.

First, we found an association between MITF targeting scores and *DCBLD2* promoter hypermethylation. *DCBLD2* has been suggested to trigger oncogenic processes in melanoma through Epidermal Growth Factor Receptor (*EGFR)* signaling [60]. This finding is also consistent with the identification of MITF as a key driver of melanoma [61, 62]. In addition, we found associations between MITF targeting and *PTPN20*, a tyrosine phosphatase coding gene, providing further evidence that disrupted signaling mediated by MITF regulation plays an important role in melanoma. Targeting by GLI1 and GLI2 was also increased in melanoma confirming their association to drug resistance in melanoma cell lines [63] and was associated with promoter hypomethylation of *MIR6893*. According to TargetScan [10], *MIR6893* regulates two related TFs, Glis Family Zinc Finger 1 and 2 (GLIS1 and GLIS2), and both have been reported to be involved in psoriasis [64], an inflammatory skin condition, which may indicate that they play a similar role in melanoma. We finally found a decrease of targeting by TBX19 to be associated with the amplification of the *HLA-DBA1* and *HLA-DQB1* genes. Both of which are known to be melanoma risk factors [65], although HLA genes are highly polymorphic and any inference on them needs to account for the underlying population structure [66]. TBX19 itself has not been implicated in melanoma, but it has been linked to lymph node metastasis in colorectal cancer [67] and TBX2, another member of the T-Box family, is involved in melanoma proliferation [68].

Second, a regression analysis identified four TFs to be strongly implicated in melanoma drug resistance. We found targeting by MITF to be negatively associated with regorafenib cell viability. This finding is consistent with studies that found MITF loss to be associated with drug resistance [69] and underscores the multifunctional role that MITF appears to play in melanoma based on our analysis. However, other studies have implicated an increased activity of MITF in resistance to BRAF inhibitor treatment [70, 71]. Another TF, ZNF778, was also a strong predictor of regorafenib cell viability (Fig. 2B, Additional file 1: Fig. S1); the ZNF778 promoter has also been found to be highly mutated in melanoma [72].

Third, we ran a transition analysis to identify TFs involved in metastasis. We found that the TFs RUNX2, GLI1, and CREB3L1 were among those with the largest differential involvement score. RUNX2 has been previously identified as a driver of epithelial to mesenchymal transition (EMT) processes and phenotype switching in melanoma [50]. CREB3LI has been reported to be activated in drug-resistant cell lines [73] and GLI1 knockout has been shown to increase sensitivity to vemurafenib [63], an approved melanoma BRAF inhibitor. Collectively, the results from these analyses suggest a co-involvement of TFs associated with both drug resistance and cell state transition in invasive disease and highlight the promise of multi-kinase targeting [74].

Extending our analysis from melanoma to all cancer types using a DRAGON multi-omic network, we correlated miRNA levels with gene dependency scores and found a negative partial correlation between *MIR664* and *GSR*, suggesting that *MIR664* post-transcriptionally regulates *GSR*. This is consistent with annotation in TargetScan database [10], which predicts *GSR* to be a target of *MIR664*, ranked 613/5387 with a total context++ score [75] (which is the sum of contributions of 14 features for each target site, lower total context++ score indicating stronger evidence) of −0.16.

Next, analyzing metabolite concentrations and protein levels, we found negative partial correlations between LDHA and glycolysis metabolites, as well as LDHA and TCA cycle metabolites. These two observations suggest an activity of LDHA in the forward direction towards lactate production through aerobic glycolysis and fermentation (Additional file 1: Fig. S2). Activation of this suboptimal pathway (Warburg effect [76]) to produce energy is a hallmark of cancer and has been correlated with poor prognosis and drug resistance [77, 78]. Because we conducted this analysis across all cell lines without filtering for a particular lineage, this result suggests that aerobic glycolysis could be prevalent in CCLE cell lines as observed in several solid tumors [79]. We also find a negative partial correlation between normalized levels of LDHA and LDHB with lactate in primary tumors and a positive partial correlation between them in metastatic tumors. This indicates that LDHA and LDHB may operate in their non-preferential direction, to control the production of lactate in metastatic tumors and its breakdown in primary tumors. It has been shown that LDHB can compensate LDHA after *LDHA* knockout [80] to produce lactate. Therefore, our network analysis identified a distinct metabolic program [81] in primary and metastatic cells, mediated by LDHA and LDHB switching. In particular, it has been shown that production of lactate drives the cellular program towards a migrating phenotype, while primary tumors may still have increased mitochondrial activity and therefore need substrates from lactate breakdown for TCA cycle [82]. Our analysis indicates that this happens by the concerted activity of both LDHA and LDHB, disrupting their physiological balance.

Examining DRAGON correlations between dabrafenib, a BRAF inhibitor and gene dependency scores, we found an association with *BRAF*, but also with *MAPK1* and *MAP2K1*. This is possibly due to compensatory mechanisms between functionally related genes [83]. In the absence of these effects, the finding makes sense because although dabrafenib is described as “selective” to BRAF [84], it has been shown to be active in cell lines with constitutively activated BRAF harboring the V600E activating mutation [85]; this subsequently triggers drug resistance by reactivating the MAPK pathway, particularly, MAPK1 and MAP2K1.

We finally analyzed the association between the levels of 2HG, an oncometabolite implicated in the hypermethylator phenotype in glioma and TF targeting to identify TFs that may be affected by changes in methylation induced by 2HG. We found significant associations between 2HG and PPARG, TP73, and GLI4 possibly mediated by promoter hypermethylation and the subsequent disruption of their binding sites. These TFs have been implicated in cancer; PPARG is linked to cell proliferation and tumor development [86], TP73 is a homologue of the tumor suppressor gene TP53, and GLI4 is an oncogene in glioma, which is among the cancer types associated with 2HG-induced epigenetic disruption.

## Conclusions

We developed netZoo as an open-source platform for the inference and analysis of GRNs including bipartite networks (inferred by PANDA, PUMA, and similar methods), multi-omic partial correlation DRAGON networks, and downstream analytical methods for community detection and differential analyses on these networks using CONDOR and MONSTER. We accomplished this by standardizing the implementations of software methods built on a common conceptual framework. This has allowed us to build a robust and reproducible codebase that we used as the core of an ecosystem of online tools for sharing of use cases through Netbooks, hosting networks in GRAND, and continued development and maintenance through ZooKeeper which is essential for software accuracy [87]. We will continue to expand netZoo (Additional file 1: Fig. S5) particularly for single-cell genomics, adding new methods [88–90] and improving implementations of existing methods, as well as building interfaces to allow methods to be combined appropriately. We will also continue to leverage the codebase to add new components in the ecosystem of online tools we developed to further aid users and developers in hosting genome-scale networks and running complex analyses on the cloud for their own investigations. Our approach has enabled an open and collaborative development model that is committed to the broad use of the methods available within netZoo and welcomes community participation in methods development by identifying errors, adding features, and discussing issues and ongoing work.

## Methods

### Applications of netZoo using the Cancer Cell Line Encyclopedia

The CCLE project characterized more than a thousand cell lines from 35 cancer types, measuring gene and miRNA expression, promoter methylation status, copy number variation, and protein and metabolite levels (Table S3). Cellular phenotypic data are available from the PRISM project on viability of these cell lines following drug exposure [91] and from cell fitness screens available through the dependency map [92]. For all analysis presented in this work, we used the following releases of CCLE data: promoter methylation data of 2018/10/22, histone marks data of 2018/11/30, miRNA expression data of 2018/11/03, metabolite levels data [38] of 2019/05/02. Cell viability assays were taken from the 19Q4 release of PRISM [91]. Cell fitness screens were taken from the 21Q1 release of project Achilles. Gene expression and copy number variation were taken from the 21Q1 release of the Dependency Map [92]. Protein levels [37] were taken from the 2020/01 version of CCLE.

Gene expression data for RNA-Seq measurements was collected on protein-coding genes and used as processed in CCLE; by log2 normalizing count data with a pseudo-count of 1. Data was available for 1376 cell lines across 19,177 genes. Methylation data was assessed using reduced representation bisulfite sequencing (RRBS) for 21,337 loci located with a 1-kb region of 17,182 genes across 843 cells, these values varied between 0 and 1. Global chromatin profiling data was assessed for 42 modified and unmodified H3 tail peptides across 897 cells and measured their abundances [36]. miRNA data consists of the quantification of the expression of 734 miRNAs across 954 cells. Metabolic data consisted of metabolite levels for 225 metabolites (124 polar and 101 lipid) using hydrophilic interaction chromatography and reversed phase chromatography in 928 cell lines [38]. Differential cell viability screens after drug exposure data consist of log fold-change of viability for 4686 compounds in 578 cells with respect to a DMSO control, as processed in PRISM [91] which corrects for batch effects and experimental confounders. CRISPR screens for the knockout of 18,119 genes across 808 cell lines describe the fitness of cell lines after gene removal. Data was used as normalized in the cancer Dependency Map [92] by removing principal components correlated to batch effects and by centering the data such as nonessential gene knockout has a value of 0, and essential knockouts have a median of −1. Gene-level copy number variation data for 18,119 genes across 808 cells was obtained by log2 normalizing the count number after adding a pseudo-count of 1; count data was derived from SNP array, whole exome or whole genome sequencing, as detailed by Ghandi and colleagues [36]. Quantitative proteomic data for 12,755 proteins was assessed in 375 cancer cell lines across 22 lineages using mass spectrometry [37]; data normalization has been described in detail by Nusinow and Gygi [93]. We processed this data by removing three low-quality samples and replacing missing entries with 0.

For the 1376 CCLE cell lines that had transcriptomic measurements, we inferred GRNs using PANDA and LIONESS algorithms (Table S4) available in netZooPy v0.8.1 and used these for various analyses. As input to PANDA network inference process, we began with a TF-to-gene prior regulatory network computed by running FIMO [15] scans of 1149 TF motifs from CIS-BP (v1.94d [5];) in the promoter region of 38,723 genes (defined as 1kb downstream of each gene’s transcription start site) in the reference human genome sequence (hg38); we adjusted the TF-gene pair by combining two previously suggested scores [94, 95]. The modified score (*s*) integrates the distance between the detected motif and the TSS with the significance of motif assignment as follows:

1 $$s\left(t,g\right)=\sum_k-{\mathit{\log}}_{10}\left(\textrm{p}-{\textrm{value}}_k\right)\ast {e}^{-\frac{d_k}{md\ast 10+1}}$$

where *t* is a transcription factor, *g* is a target gene, *k* is the number of binding sites of *t* identified in the promoter region of *g*, *d*_*k*_ denotes the distance of *t*’s biding site *k* to TSS of *g*, *md* the median of all the distances *d*, and p-value_*k*_ the significance of assignment of binding site *k*. Therefore, the TF motif network associates 1149 TFs to 38,723 target genes. In addition, we used as inputs a TF PPI network derived from the STRING database [2] (using the aggregate score for human interactions only and scaling them between 0 and 1) that we restricted to a list of 1603 TFs as defined by Lambert and colleagues [5], and a gene coexpression network between 19,177 protein-coding genes across 1376 cells. The latter network is a Pearson correlation network based on RNA-Seq data as preprocessed in CCLE by adding a pseudo-count of 1 to TPM gene expression data and applying log2 transformation. The resulting PANDA network includes regulatory associations between 1132 TFs and 18,560 genes because we set the “mode” parameter in PANDA to “intersection” which takes the intersecting TFs and genes between the three input networks. Then, we used LIONESS to infer regulatory networks for each of the 1376 cell lines; all networks can be found in the GRAND database (https://grand.networkmedicine.org/cell/).

We also computed TF targeting scores [43] by computing the weighted outdegree for each TF in each cell line-specific network. TF targeting scores as a network metric could be interpreted as the number of target genes that each TF has and therefore reflects the activity of TFs in various contexts. We showed that building differential TF targeting scores by comparing TF targeting scores between conditions allows to identify newly acquired target genes in a case versus control setting. We found that TF targeting scores [43] and differential targeting scores [35] summarize accurately biological processes in the cell such as those activated in cancer and as a response to drug exposure.

### TF targeting analysis

To find associations between TF targeting and promoter methylation status and copy number variation status, we selected 76 melanoma CCLE cell lines and we computed the significance of associations using ANOVA as implemented in the Python package statsmodels v0.13.2 [96]. Since we were mostly interested in finding strong associations and prominent regulatory hallmarks of melanoma, we discretized the input data by considering a gene to be amplified if it had more than three copies and to be deleted if both copies are lost. For promoter methylation data, promoters were defined in CCLE as the 1kb region downstream of the gene’s transcriptional start site (TSS). We defined hypermethylated promoter sites as those having methylation status with a *z*-score greater than three and we defined hypomethylated sites as those having methylation status with a *z*-score less than negative three; we considered a gene to be amplified if it had evidence of more than three copies in the genome and to be deleted if both copies are lost. We only computed the associations if they had at least three positive instances of the explanatory variable (for example, for a given gene at least three cell lines had a hypomethylation in that gene’s promoter) and corrected for multiple testing using a false discovery rate of less than 25% following the Benjamini-Hochberg procedure [97].

In all melanoma cell lines, for each modality (promoter hypomethylation, promoter hypermethylation, gene amplification, and gene deletion) and for each gene, we built an ANOVA model using TF targeting as the response variable across all melanoma cell lines while the status of that gene (either promoter methylation or copy number status) was the explanatory variable. For example, in modeling promoter hypermethylation, we chose positive instances to represent hypermethylated promoters and negative instances for nonmethylated promoters along with an additional factor correcting for the cell lineage. Similarly, for copy number variation analysis, we chose positive instance to represent amplified genes and negative instances for nonamplified genes while correcting for cell lineage. We only computed the associations if they had at least three positive instances of the explanatory variable (for example, promoter hypomethylation in at least three cell lines).

To predict drug response using TF targeting, we conducted a linear regression with elastic net [45] regularization as implemented in the Python package sklearn v1.1.3 using an equal weight of 0.5 for L1 and L2 penalties using regorafenib cell viability assays in melanoma cell lines as a response variable and the targeting scores of 1,132 TFs (Table S5) as the explanatory variable.

Finally, to model EMT in melanoma, we used MONSTER on two LIONESS networks of melanoma cancer cell lines, one representing a primary tumor (Depmap ID: ACH-000580) as the initial state and the other a metastasis cell line (Depmap ID: ACH-001569) as the end state. We modified the original implementation of MONSTER that implements its own network reconstruction procedure to take any input network, such as LIONESS networks. MONSTER identifies differentially involved TFs in the transition by shuffling the columns of the initial and final state adjacency matrices 1000 times to build a null distribution, which is then used to compute a standardized differential TF involvement score by scaling the obtained scores by those of the null distribution.

### Computing CCLE multi-omic associations

We used DRAGON to compute partial correlations between multi-omic data of CCLE cell lines. In particular, we computed partial correlations between the four following data type pairs across all CCLE cell lines: (1) miRNA levels and gene knockout screens, (2) protein levels and metabolite levels, (3) cell viability assays after drug exposure and gene knockout screens, and (4) TF targeting and metabolite levels. For each association, the final number of cell line samples is the intersection of the cell lines for each modality. DRAGON builds a GGM that implements covariance shrinkage with tuning parameters specific to each biological layer or “ome,” represented by a different data type, a novel addition to covariance shrinkage that enables DRAGON to account for varying data structures and sparsity of different multi-omic layers [52]. The magnitude of DRAGON partial correlation values may not be always interpretable without a reference because they are derived from a regularized, shrunken covariance matrix [98]. All variables were standardized to have a mean of 0 and a standard deviation of 1 before running DRAGON.

To compute associations between protein levels and metabolite concentrations, we averaged protein isoform levels to reduce the set of 12,755 measured proteins to 12,197 unique proteins. The final number of samples used to compute this association represented 258 cells shared between the 375 cells for proteomics data and 928 cells for metabolomic data. To compute associations between LDH levels and its substrate lactate, and because the LDH isozymes (LDHA and LDHB) catalyze opposite biochemical reactions, we created two new variables in the DRAGON network accounting for the ratio between isozymes:

$${\displaystyle \begin{array}{c}{\textrm{LDHA}}_{\textrm{normalized}}={1}_{\left[\frac{\textrm{LDHA}}{\textrm{LDHB}}>1\right]}.\frac{\textrm{LDHA}}{\textrm{LDHB}}\\ {}{\textrm{LDHB}}_{\textrm{normalized}}={1}_{\left[\frac{\textrm{LDHB}}{\textrm{LDHA}}>1\right]}.\frac{\textrm{LDHB}}{\textrm{LDHA}}\end{array}}$$

where LDHA and LDHB represent protein levels of LDH isozymes. This normalization reflects our understanding of the nonlinear relation between the ratio of LDHA/LDHB and lactate concentrations: when LDHA is dominant, LDH produces lactate; therefore, we expect a positive correlation with lactate levels, and conversely, when LDHB is dominant, lactate is a substrate for LDH and the correlation should be negative. We did not include pyruvate concentrations because it was not among the measured metabolites in CCLE.

### CCLE pan-cancer map

To enable further exploration and discovery of biological associations, we built an online resource representing a multi-tiered regulatory network. First, to build a pan-cancer multi-tiered network that connects the genotype to cellular phenotypes, we extended DRAGON networks from modeling pairwise interactions between two biological variables to a multi-omic network that includes more than two node types by sequentially adding a new layer to an initial pairwise DRAGON network. In addition, since DRAGON networks are undirected, we added direction based on our understanding of how biological elements interact with each other. For example, gene expression nodes are upstream of protein level nodes and metabolite nodes. To facilitate browsing and limit exploration to potentially causal associations that best reflect our understanding of how different data types link to one another in cellular biology, our approach was to prune edges between the same node type to build bipartite DRAGON networks between each pair of genomic modalities. In particular, promoter methylation status, copy number variation, histone marks, and miRNA were linked to gene expression in a pairwise fashion. Then, gene expression was linked to protein levels, which in turn was associated with cellular phenotypes represented by metabolite levels, drug sensitivity, and cell fitness following CRISPR gene knockout. To reduce the size of the network to the most relevant positive and negative associations, only the 2000 most positive correlations and the 2000 most negative correlations in each pairwise association in each of the bipartite networks were retained in the final multi-omic network. The CCLE online pan-cancer map was built using Vis.js (v8.5.2) and can be queried for biological associations using user input queries at https://grand.networkmedicine.org/cclemap.

### Software package

All analyses were performed using netZooPy v0.8.1, the Python distribution of the netZoo (netzoo.github.io). NetZoo methods are implemented in R, Python, MATLAB, and C. netZooR v1.3 is currently implemented in R v4.2 and available through GitHub (https://github.com/netZoo/netZooR) and Bioconductor (https://bioconductor.org/packages/netZooR) and includes PANDA, LIONESS, CONDOR, MONSTER, ALPACA, PUMA, SAMBAR, OTTER, CRANE, SPIDER, EGRET, DRAGON, and YARN. netZooPy v0.8.1 is implemented in Python v3.9 and includes PANDA, LIONESS, CONDOR, PUMA, SAMBAR, OTTER, and DRAGON. netZooM v0.5.2 is implemented in MATLAB 2020b (The Mathworks, Natick, MA, USA) and includes PANDA, LIONESS, PUMA, OTTER, and SPIDER. netZooC v0.2 implements PANDA and PUMA.

## Supplementary Information

**Additional file 1: Text S1.** Summary of netZoo methods. **Figure S1.** Elastic net coefficients of regorafenib drug sensitivity regression on TF targeting. The analysis includes all 1,132 TFs modeled in the GRNs of 76 melanoma cell lines. The tails of this distribution are represented in Fig. 2B. **Figure S2.** Correlating TCA cycle metabolite and enzyme levels to infer pathway direction. **Figure S3.** Absolute LDH protein levels do not convey the underlying metabolic network. **Figure S4.** Reconstruction of a multi-omic partial correlation network using DRAGON. **Figure S5.** Timeline of netZoo methods’ publications. **Table S1.** Input data for netZoo methods. **Table S2.** Pairwise combinations of multi-omic data to build a CCLE integrated partial correlation network. **Table S3.** Resources table. Data used for the various analysis presented in the main text is presented in the following table. **Table S4.** Experimental design and statistical methods for the analyses presented in the main text. **Table S5.** Genes names of 1,132 TFs modeled in CCLE GRNs.

**Additional file 2.** Review history.

## Data Availability

netZoo methods are available at https://netzoo.github.io under GPL-3.0 open-source license for netZooR [99], netZooPy [100], netZooM [101], and netZooC [102]. CCLE cell line GRNs can be downloaded at https://grand.networkmedicine.org/cell/ and the CCLE multi-tiered map can be accessed at https://grand.networkmedicine.org/cclemap/. Code to reproduce the analyses presented in the paper is available through Netbooks (https://netbooks.networkmedicine.org). For gene regulatory network analysis, we used the following data sets: DepMap v21Q1 [103], STRINGdb v11.0 [104], and CIS-BP 1.94d [105]. For the multi-omic CCLE network inference analysis, we used gene expression and copy number variation data from DepMap v21Q1 [103], promoter methylation from CCLE v2018/10/22 [106], histone marks from CCLE v2018/11/30 [107], miRNA expression data from CCLE v2018/11/03 [108], metabolite levels from CCLE v2019/05/02 [109], drug viability assays from PRISM v19Q4 [110], CRISPR screens data from Project Achilles v21Q1 [111], and protein levels from CCLE v2020/01 [112].
