# Supplementary material for: The Network Zoo: a multilingual package for the inference and analysis of gene regulatory networks
Source: Genome Biol. 2023 Mar 9;24:45. doi: 10.1186/s13059-023-02877-1 (PMC9999668; doi:10.1186/s13059-023-02877-1)
Supplement: Supplementary file 1 — Additional file 1: Text S1. Summary of netZoo methods. Figure S1. Elastic net coefficients of regorafenib drug sensitivity regression on TF targeting. The analysis includes all 1,132 TFs modeled in the GRNs of 76 melanoma cell lines. The tails of this distribution are represented in Fig. 2B. Figure S2. Correlating TCA cycle metabolite and enzyme levels to infer pathway direction. Figure S3. Absolute LDH protein levels do not convey the underlying metabolic network. Figure S4. Reconstruction of a multi-omic partial correlation network using DRAGON. Figure S5. Timeline of netZoo methods’ publications. Table S1. Input data for netZoo methods. Table S2. Pairwise combinations of multi-omic data to build a CCLE integrated partial correlation network. Table S3. Resources table. Data used for the various analysis presented in the main text is presented in the following table. Table S4. Experimental design and statistical methods for the analyses presented in the main text. Table S5. Genes names of 1,132 TFs modeled in CCLE GRNs. [file 13059_2023_2877_MOESM1_ESM.docx]

**Supplementary information for**

**The Network Zoo: a multilingual package for the inference and analysis of gene regulatory networks**

Marouen Ben Guebila^1^, Tian Wang^1,2^, Camila M. Lopes-Ramos^1,3^, Viola Fanfani^1^, Deborah Weighill^1,4^, Rebekka Burkholz^1,5^, Daniel Schlauch^1,6^, Joseph N. Paulson^7^, Michael Altenbuchinger^1,8^, Kate H. Shutta^1,3^, Abhijeet R. Sonawane^3,9^, James Lim^10,11^, Genis Calderer^12^, David van Ijzendoorn^13,14^, Daniel Morgan^3,15^, Alessandro Marin^16^, Cho-Yi Chen^1,17,18^, Qi Song^3,19^, Enakshi Saha^1^, Dawn L. DeMeo^3^, Megha Padi^10^, John Platig^3^, Marieke L. Kuijjer^12,13,20^, Kimberly Glass^1,3^, John Quackenbush^1,3,17^

^1^Department of Biostatistics, Harvard T.H. Chan School of Public Health, Boston, MA, USA

^2^Present address: Biology Department, Boston College, Chestnut Hill, MA, USA

^3^Channing Division of Network Medicine, Brigham and Women's Hospital and Harvard Medical School, Boston, MA, USA

^4^Present address: Lineberger Comprehensive Cancer Center, University of North Carolina at Chapel Hill, NC, USA

^5^Present address: CISPA Helmholtz Center for Information Security, Saarbrücken, Germany

^6^Present address: Genospace, LLC, Boston, MA, USA

^7^Department of Biochemistry and Molecular Biology, Pennsylvania State University College of Medicine, Hershey, PA, USA.

^8^Present address: Department of Medical Bioinformatics, University Medical Center Göttingen, Göttingen, Germany

^9^Present address: Center for Interdisciplinary Cardiovascular Sciences, Division of Cardiovascular Medicine, Department of Medicine, Brigham and Women’s Hospital, Boston, MA, USA

^10^Department of Molecular and Cellular Biology, University of Arizona, Tucson, AZ, USA

^11^Present address: Monoceros Biosystems, LLC, San Diego, CA, USA

^12^Center for Molecular Medicine Norway, Nordic EMBL Partnership, University of Oslo, Oslo, Norway

^13^Department of Pathology, Leiden University Medical Center, Leiden, The Netherlands

^14^Present address: Department of Pathology, Stanford University School of Medicine, CA, USA

^15^Present address: Hong Kong University, School of Biomedical Sciences, Honk Kong

^16^Expert Analytics AS, Oslo, Norway

^17^Dana-Farber Cancer Institute, Boston, MA, USA

^18^Present address: Institute of Biomedical Informatics, National Yang Ming Chiao Tung University, Taipei 112, Taiwan

^19^Present address: Computational Biology Department, Carnegie Mellon University, Pittsburgh, PA, USA

^20^Leiden Center for Computational Oncology, Leiden University, Leiden, The Netherlands

**Supplementary text**

**Text S1: Summary of netZoo methods**

Passing Attributes between Networks for Data Assimilation (PANDA) [14] is a method for estimating bipartite gene regulatory networks (GRNs) consisting of two types of nodes: transcription factors (TFs) and genes. An edge between TF i and gene j indicates that gene j is regulated by TF i. The edge weight represents the strength of evidence for this regulatory relationship obtained by integrating three types of biological data: gene expression data, protein-protein interaction (PPI) data, and transcription factor binding motif (TFBM) data. PANDA is an iterative approach that begins with a seed GRN estimated from TFBMs and uses message passing between data types to refine the seed network to a final GRN that is consistent with the information contained in gene expression, PPI, and TFBM data.

Linear Interpolation to Obtain Network Estimates for Single Samples (LIONESS) [20] is a flexible method for single-sample network integration. The machinery behind LIONESS is a leave-one-out approach. To construct a single-sample network for sample i, a first network is estimated on the full dataset and a second network is estimated on the dataset with sample i withheld. The single-sample network is then estimated based on the difference between these two networks. Any method that can be used to estimate a network can be used with LIONESS to estimate single-sample networks. Two common use cases are the use of LIONESS to generate single-sample GRNs based on PANDA and the use of LIONESS to generate single-sample Pearson correlation networks.

COmplex Network Description Of Regulators (CONDOR) [24] is a tool for community detection in bipartite networks. Many community detection methods for unipartite networks are based on the concept of maximizing a modularity metric that compares the weight of edges within communities to the weight of edges between communities, prioritizing community assignments with higher values of the former relative to the latter. CONDOR extends this concept to bipartite networks by optimizing a bipartite version of modularity defined by Barber [25]. To enable bipartite community detection on large networks such gene regulatory networks, CONDOR uses a fast unipartite modularity maximization method on one of the two unipartite projections of the bipartite network. In Platig and colleagues [24] , CONDOR is applied to bipartite networks of single nucleotide polymorphisms (SNPs) and gene expression, where a network edge from a SNP node to a gene node is indicative of an association between the SNP and the gene expression level, commonly known as an expression quantitative trait locus (eQTL). Communities detected with CONDOR contained local hub nodes ("core SNPs") enriched for association with disease, suggesting that functional eQTL relationships are encoded at the community level.

Modeling Network State Transitions from Expression and Regulatory data (MONSTER) [28] is a method for estimating transitions between network states by modeling the adjacency matrix of one state as a linear transformation of the adjacency matrix of another. Like LIONESS, MONSTER is a flexible method that does not require a particular type of network structure. MONSTER models the perturbation of an initial network A into a perturbed network B according to a matrix product B = AT. T is a transition matrix encoding the changes that map A to B. When A and B are gene regulatory networks, i.e., bipartite networks between TFs and genes, the MONSTER framework leads naturally to the definition of TF involvement as the sum of the off-diagonal weights for a transcription factor i in the transition matrix T. This perspective enables MONSTER to identify differentially involved TFs that contribute to network transitions differently between different conditions. This dimension cannot be captured from a traditional differential expression analysis of TFs, which will not detect TFs that have the same concentration between conditions.

ALtered Partitions Across Community Architectures (ALPACA) [26] is a method for differential network analysis that is based on a novel approach to comparison of network community structures. Comparisons of community structure have typically been accomplished by assessing which nodes switch community membership between networks ("community comparison") or by computing the edge weight differences by subtracting the adjacency matrices of two networks and then performing community detection on the resulting differential network ("edge subtraction"). Both these approaches have important limitations. Community comparison is subject to a resolution limit and cannot detect differences smaller than the average community size in a network. Edge subtraction transfers noise from both of the original networks to the differential network, leading to an imprecise estimator. Moreover, positive and negative edge differences cannot be distinguished in the subsequent community detection performed on the differential network.

In contrast to community comparison and edge subtraction, ALPACA compares the community structure of two networks by optimizing a new metric: "differential modularity". In the ALPACA algorithm, one network is defined as the reference network and the second is defined as the perturbed network. The differential modularity metric measures the extent to which edges in a community in the perturbed network differ from those that would be expected by random chance according to a null distribution based on the reference network. Community structure of the perturbed network is determined by maximizing this differential modularity. The resulting communities are "differential modules" that show how the perturbed network differs from the reference network at the community level.

PANDA Using MicroRNA Associations (PUMA) [19] extends the PANDA framework to model how microRNAs (miRNAs) participate in gene regulatory networks. PUMA networks are bipartite networks that consist of a regulatory layer and a layer of genes being regulated, similar to PANDA networks. While the regulatory layer of PANDA networks consists only of transcription factors (TFs), the regulatory layer of PUMA networks consists of both TFs and miRNAs. A PUMA network is seeded using a combination of input data sources such as motif scans or ChIP-seq data (for TF-gene edges) and an miRNA target prediction tool such as TargetScan or miRanda (for miRNA-gene edges). PUMA uses a message passing framework similar to PANDA to integrate this prior information with gene-gene coexpression and protein-protein interactions to estimate a final regulatory network incorporating miRNAs. Kuijjer and colleagues [19] apply PUMA to 38 GTEx tissues and demonstrate that PUMA can identify important patterns in tissue-specific regulation of genes by miRNA.

Subtyping Agglomerated Mutations By Annotation Relations (SAMBAR) [29] is a tool for studying cancer subtypes based on patterns of somatic mutations in curated biological pathways. Rather than characterize cancer according to mutations at the gene level, SAMBAR agglomerates mutations within pathways to define a pathway mutation score. To avoid bias based on pathway representation, these pathway mutation scores correct for the number of genes in each pathway as well as the number of times each gene is represented in the universe of pathways. By taking a pathway rather than gene-by-gene lens, SAMBAR both de-sparsifies somatic mutation data and incorporates important prior biological knowledge. Kuijjer and colleagues [29] demonstrate that SAMBAR is capable of outperforming other methods for cancer subtyping, producing subtypes with greater between-subtype distances; the authors use SAMBAR for a pan-cancer subtyping analysis that identifies four diverse pan-cancer subtypes linked to distinct molecular processes.

Optimization to Estimate Regulation (OTTER) [16] is a GRN inference method based on the idea that observed biological data (PPI data and gene co-expression data) are projections of a bipartite GRN between TFs and genes. Specifically, PPI data represent the projection of the GRN onto the TF-TF space and gene co-expression data represent the projection of the GRN onto the gene-gene space. OTTER reframes the problem of GRN inference as a problem of relaxed graph matching and finds a GRN that has optimal agreement with the observed PPI and coexpression data. The OTTER objective function is tunable in two ways: first, one can prioritize matching the PPI data or the coexpression data more heavily depending on one's confidence in the data source; second, there is a regularization parameter that can be applied to induce sparsity on the estimated GRN. The OTTER objective function can be solved using spectral decomposition techniques and gradient descent; the latter is shown to be closely related to the PANDA message-passing approach.

Constrained Random Alteration of Network Edges (CRANE) [27] is a method for determining statistical significance of structural differences between networks. Analysis with CRANE is a four-phase process. The first step of CRANE is to estimate two networks: a reference network and a perturbed network. In the same spirit as LIONESS, CRANE is flexible: any network inference method (e.g., correlation, partial correlation, PANDA) can be used at this stage. In the second step, differential features are determined by comparing the reference and perturbed networks. Here, CRANE is again flexible: such differential features could arise from simple measures such as a comparison of node degree or centrality, or from more nuanced techniques such as differential module detection with ALPACA. Third, a large number of constrained random networks are developed based on the network structure of the reference network. By comparing each random network with the original reference network, a set of null differential measures is obtained. Fourth, the observed differential features from step two can be compared with the null distribution from step three to generate empirical p-values. A typical workflow for applying CRANE in netZooR would involve fitting PANDA networks in step one and using ALPACA to estimate differential modules in step two.

Seeding PANDA Interactions to Derive Epigenetic Regulation (SPIDER) [17] extends the PANDA framework by incorporating DNase-Seq data to account for chromatin state for the prediction of TF binding sites. The method consists of processing DNase-Seq data to find open chromatin regions and build a “mask” matrix that is then overlaid on the TF-gene motif network to select binding sites that are available fro TF binding. This method can be applied for various biological contexts such as cell lines and tissues. Sonawane and colleagues [17] have employed their method to model cell- type specific GRNs using DNase-Seq data from ENCODE and showed that integrating epigenetic data in SPIDER networks allows building more accurate networks.

Estimating the Genetic Regulatory effects on TFs (EGRET) [18] incorporates genetic variants as a fourth data type in the PANDA message-passing framework, enabling the estimation of genotype-specific GRNs. Genetic variants can alter transcription factor binding by affecting the composition of motif sites on the DNA. Not every genetic variant has such an affect; EGRET incorporates only genetic variants which have (1) been shown to be associated with gene expression (expression quantitative trait loci, or eQTL), and (2) are predicted to affect transcription factor binding based on a tool called QBiC. This information is used in combination with TFBM predictions as input to the PANDA message-passing framework. The resulting EGRET network is a genotype-specific bipartite GRN that is similar to a PANDA network but incorporates the information contained by individual genetic variation.

Determining Regulatory Associations using Graphical models on Omics Networks (DRAGON) [21] is a method for estimating multiomic Gaussian graphical models (GGMs, also known as partial correlation networks) that incorporate two different omics data types. DRAGON builds off of the popular covariance shrinkage method of Ledoit and Wolf [98] with an optimization approach that explicitly accounts for the differences in two separate omics "layers" in the shrinkage estimator. The resulting sparse covariance matrix is then inverted to obtain a precision matrix estimate and a corresponding GGM. Although GGMs assume normally distributed data, DRAGON can be used on any type of continuous data by transforming data to approximate normality prior to network estimation. Currently, DRAGON can be applied to estimate networks with two different types of omics data. Investigators interested in applying DRAGON to more than two types of omics data can consider estimating pairwise networks and "chaining" them together.

Yet Another RNa-seq package (YARN) [30] is a package that combines quality control, gene filtering, and normalization steps to streamline the preprocessing of large-scale, multi-tissue gene expression data from resources such as the Genotype-Tissue Expression (GTEx) project. Among other steps, YARN uses principal coordinate analysis (PCoA) to determine if samples collected from different sites on the same tissue (for example, transverse and sigmoid colon) can be treated as "transcriptionally indistinguishable" and grouped together to increase power for downstream analyses. Paulsson and colleagues [30] demonstrate the use of YARN to develop a pan-cancer RNA-seq dataset for 30,333 genes from 9435 samples across 38 tissues from the GTEx dataset.

**Supplementary figures**


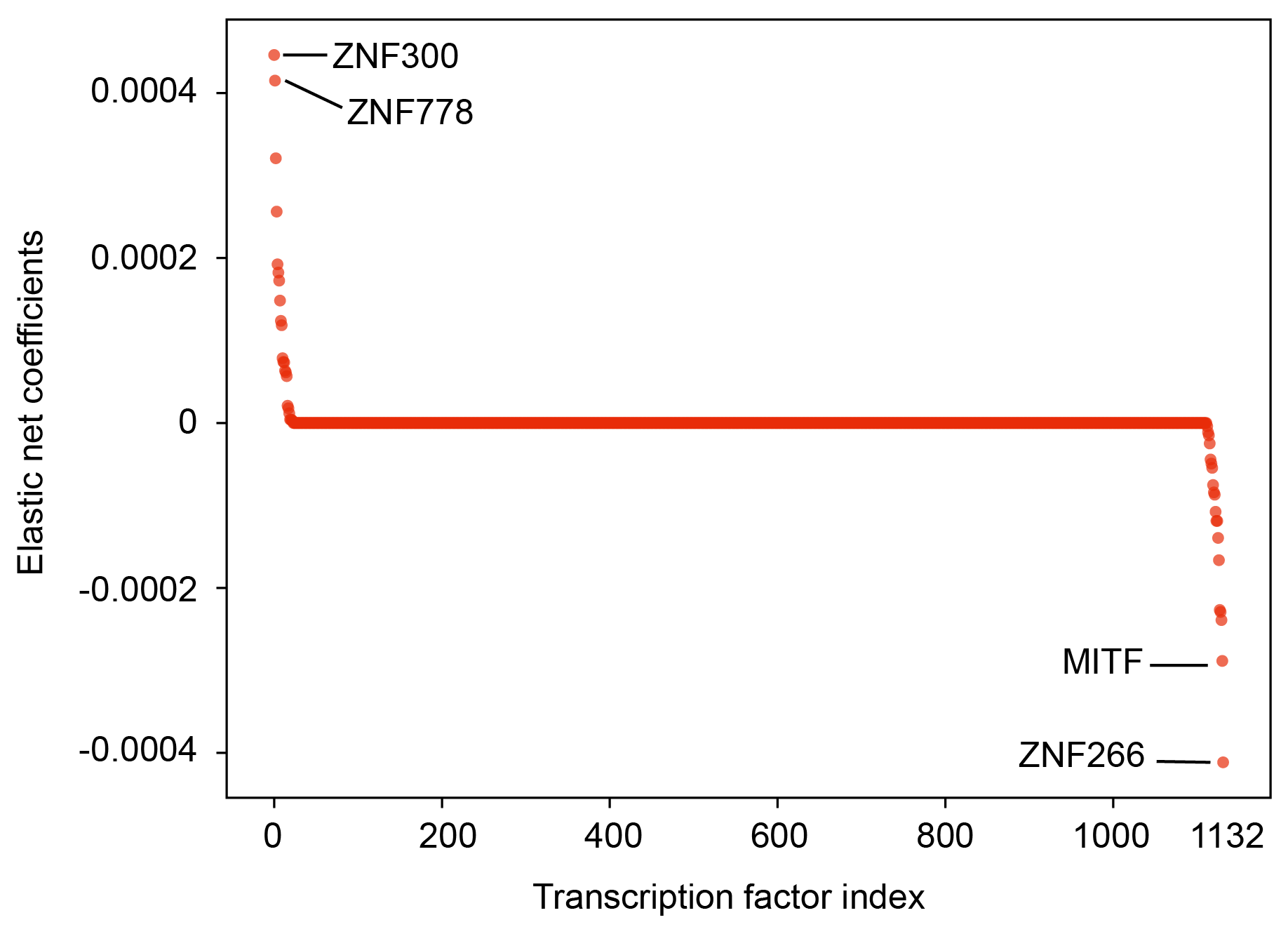


**Figure S1** Elastic Net coefficients of Regorafenib drug sensitivity regression on TF targeting. The analysis includes all 1,132 TFs modeled in the GRNs of 76 melanoma cell lines. The tails of this distribution are represented in Figure 2B.


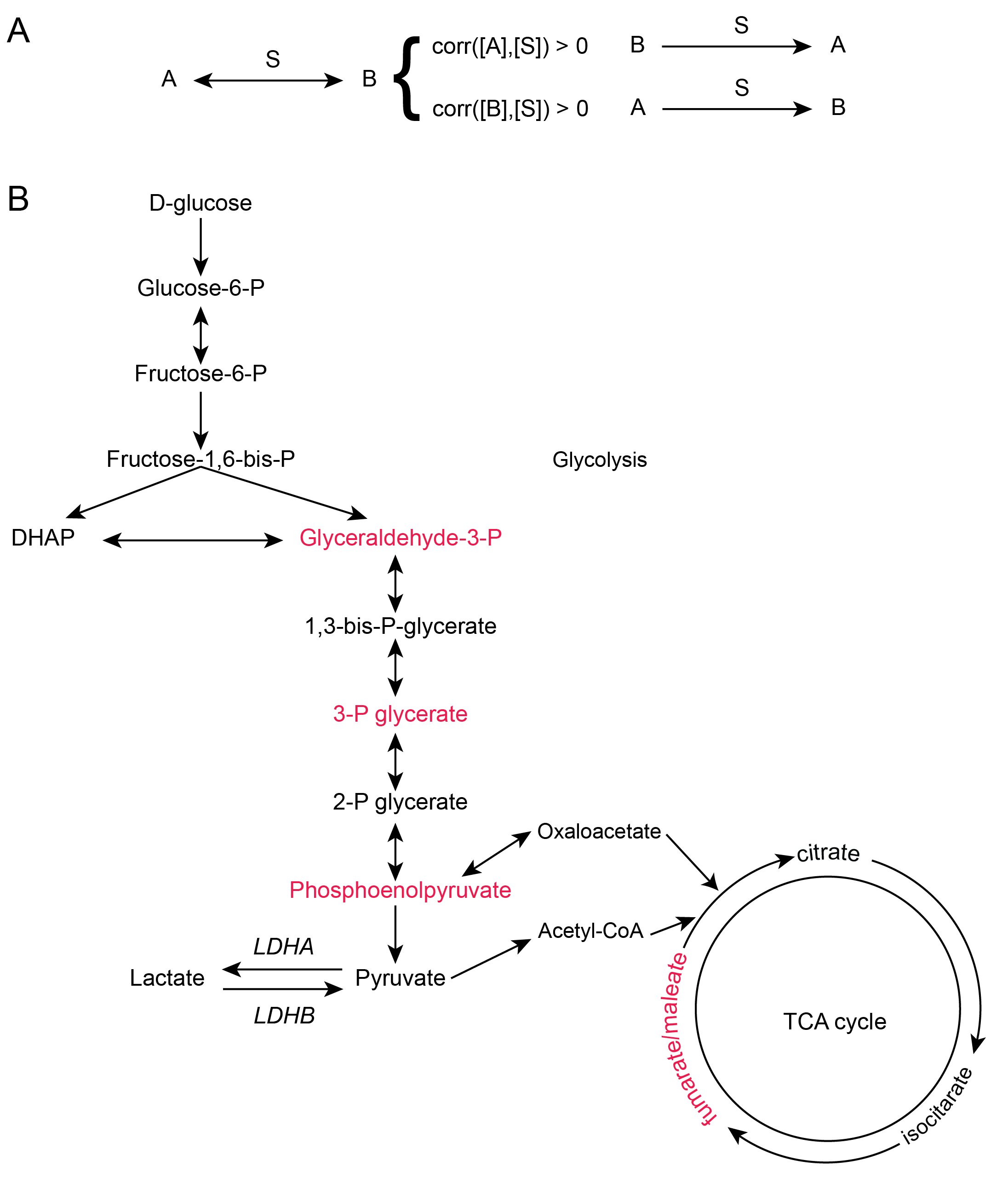


**Figure S2** Correlating TCA cycle metabolite and enzyme levels to infer pathway direction. **A** Fundamental relation between metabolite levels and enzyme concentrations. The direction of a biochemical reaction can be inferred by correlating metabolite and enzyme levels. **B** Predicted glycolysis and TCA cycle directions based on metabolite and enzyme levels. Red-colored metabolites are negatively correlated with LDHA enzyme levels in CCLE cell lines which suggests that Glycolysis operates in the forward direction and that TCA cycle does not break down the product of glycolysis, thereby a switch to lactate production pathway.


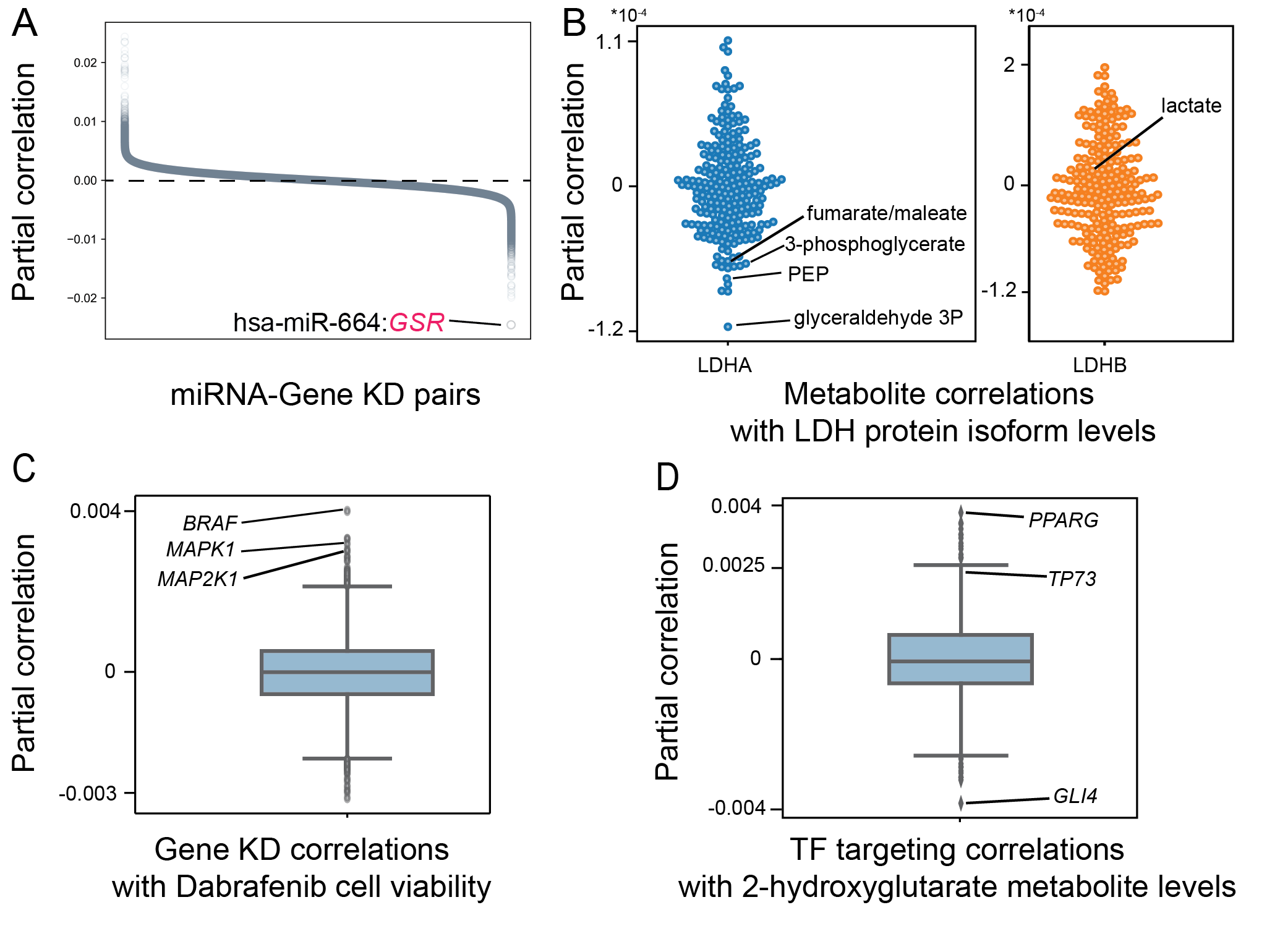


**Figure S3** Absolute LDH protein levels do not convey the underlying metabolic network. **A** Partial correlation of LDHB protein levels and various metabolites using DRAGON. Without accounting for LDHA levels, the isozyme that catalyzes the reverse biochemical reactions to produce lactate, LDHB and lactate levels are positively correlated, which may indicate that lactate is produced by LDHB, which is in fact a spurious correlation. This is due to the fact that **B** LDHA and LDHB levels are positively correlated (Pearson’s r=0.36) and therefore cell lines that have high levels of LDHB produce pyruvate but tend to have high levels of LDHA converting pyruvate back to lactate, which may falsely indicate that LDHB produces lactate (A). **C** Normalizing LDHA by the levels of its isozyme LDHB allows to recover the known canonical relationship with lactate production in CCLE cell lines. **D** LDHA and LDHB canonical metabolic cycle.


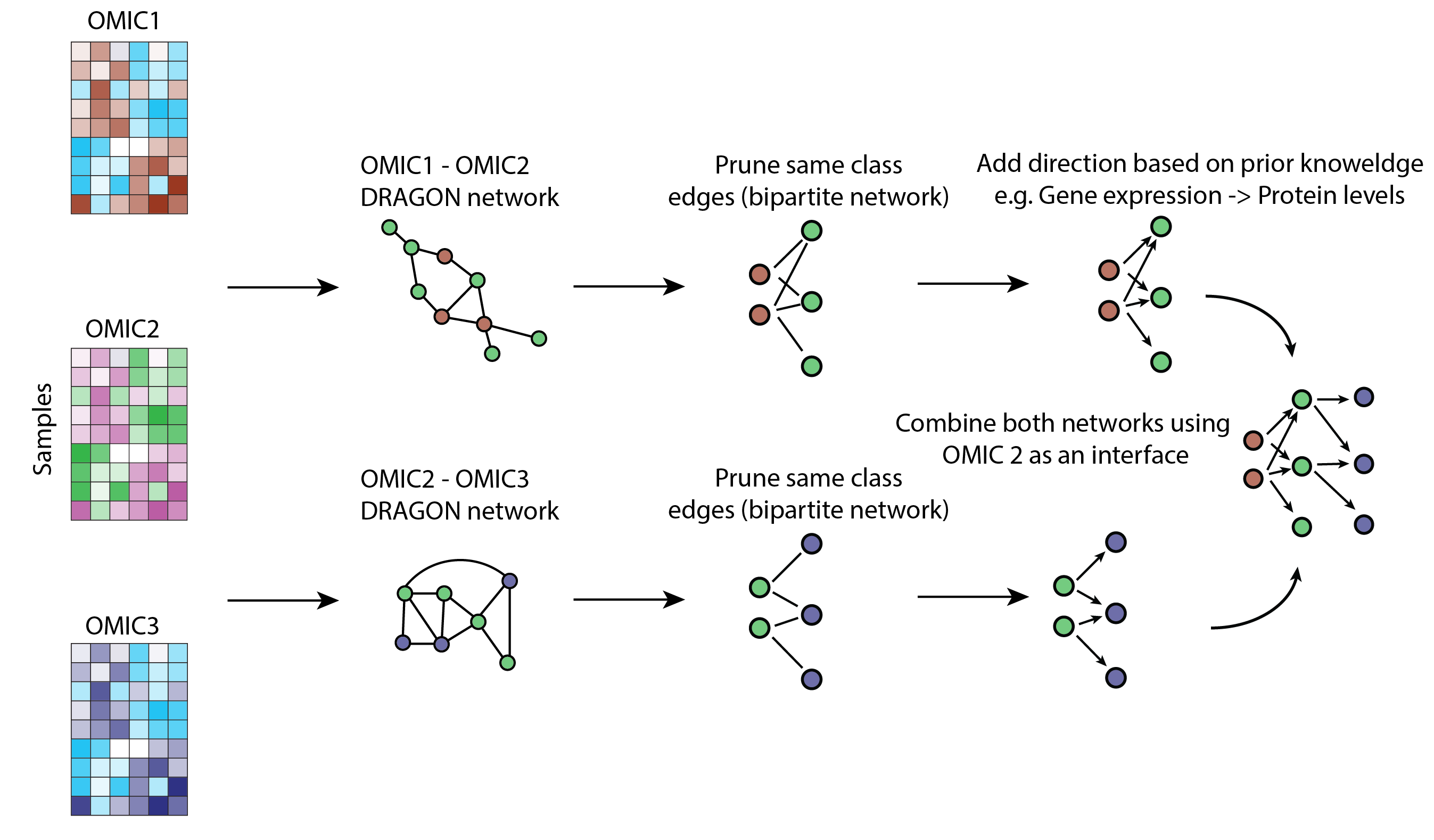


**Figure S4** Reconstruction of a multi-omic partial correlation network using DRAGON. The example illustrates the creation of a tripartite network. DRAGON generates unipartite undirected network between two pairs of “omics”. To reflect prior biological knowledge, we prune same class edges and add edges directions. Then, we combine two networks by using the intersecting ‘omic’ as an interface.


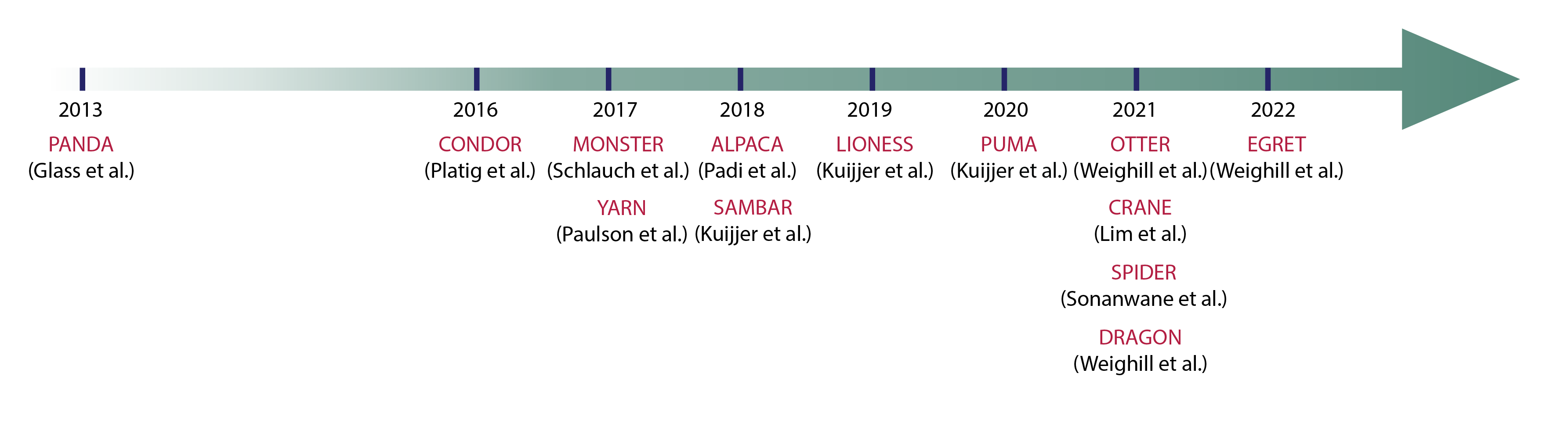
**Figure S5** Timeline of netZoo methods’ publications.

**Supplementary tables**

**Table S1** – Input data for netZoo methods.

| Group | Method | Description | Input 1 | Input 2 | Input 3 | Input 4 |
| --- | --- | --- | --- | --- | --- | --- |
| Data processing | YARN | Tissue-aware transcriptomic data processing and normalization | Gene expression data from RNA-Seq experiments and their tissue assignment | | | |
| Network reconstruction | PANDA | TF-gene cis-regulatory network inference | TF-gene motif network as a prior, e.g., using CIS-BP | TF PPI network, e.g., from STRINGdb | Gene coexpression network | |
|  | PUMA | miRNA-gene post-transcriptional regulatory network inference | miRNA-gene network as a prior, e.g., using miRNA target prediction tool such as TargetScan | | Gene coexpression network | |
|  | SPIDER | DNA accessibility-informed TF-gene cis-regulatory network inference | TF-gene motif network as a prior | TF PPI network, e.g., from STRINGdb | Gene coexpression network | Chromatin accessibility data (DNase-Seq) |
|  | EGRET | Genotype-specific TF-gene cis-regulatory network inference | TF-gene motif network as a prior | TF PPI network, e.g., from STRINGdb | Gene coexpression network | Genotype data (SNPs) to estimate cis-eQTLs |
|  | DRAGON | Inference of partial correlation network between two omic data types that may have different structures or properties | Any omic data e.g., RNA-Seq, Methylation status, Copy number variation, chromatin accessibility | | Any other data possibly co-measured in the same samples as omic data 1 and that generally represents a phenotype (disease score, response to drug) but also another omic data, e.g., protein and metabolite levels | |
|  | OTTER | TF-gene cis-regulatory network inference using graph matching | TF-gene cis-regulatory network inference | TF-gene motif network as a prior | TF PPI network | Gene coexpression network |
|  | LIONESS | Single-sample network reconstruction | Aggregate gene regulatory network, e.g., PANDA, across samples in a population. | | RNA-Seq data for several samples in a population. | |
| Community detection | CONDOR | Community detection in bipartite graphs | Gene regulatory network that has a bipartite structure, e.g., a TF- gene PANDA or OTTER network. | | | |
|  | ALPACA | Differential community detection | A first gene regulatory network that represent a case, e.g., a PANDA cancer network. | | A second gene regulatory network that represent a control, e.g., a PANDA network in matching normal tissue. | |
|  | CRANE | Assessment of significance of disease modules using biological constraints | A first gene regulatory network that represent a control, e.g., a PANDA or coexpression network in a normal tissue. | | A second gene regulatory network that represent a case, e.g., a PANDA or coexpression network in matching cancer tissue. | |
| Differential analysis | MONSTER | Estimation of regulatory drivers of transition between an initial and a final regulatory network | An initial gene regulatory network that represent a control, e.g., a PANDA or coexpression network in a normal tissue. | | A final gene regulatory network that represent a case, e.g., a PANDA or coexpression network in matching cancer tissue. | |
| Network annotation | SAMBAR | Patient-specific inference of active pathways in cancer using somatic mutation data | Genotype data for a group of patients (somatic mutations load across genes for a group of patients) | | Pathway annotation for human genes, e.g., Gene Ontology | |

**Table S2** – Pairwise combinations of multi-omic data to build a CCLE integrated partial correlation network.

| Pair | Omic 1 | Omic 2 |
| --- | --- | --- |
| 1 | Promoter methylation | Gene expression |
| 2 | Copy number variation | Gene expression |
| 3 | Histone marks | Gene expression |
| 4 | miRNA expression | Gene expression |
| 5 | Gene expression | Protein levels |
| 6 | Protein levels | Metabolite levels |
| 7 | Protein levels | Drug sensitivity (Cell viability) |
| 8 | Protein levels | Cell fitness (CRISPR KO) |

**Table S3 –** Resources table**.** Data used for the various analysis presented in the main text is presented in the following table.

| Data type | Analysis | Source | Link | Reference |
| --- | --- | --- | --- | --- |
| Gene expression | Gene regulatory network reconstruction in CCLE cell lines | DepMap v21Q1 | https://depmap.org/portal/download/ | [92] |
| Protein-protein interaction network |  | STRINGdb v11 | https://string-db.org/ | [2] |
| Motif network |  | CIS-BP v1.94d | http://humantfs.ccbr.utoronto.ca/ | [5] |
| Gene expression | Multi-omic CCLE network inference | DepMap v21Q1 | https://depmap.org/portal/download/ | [92] |
| Copy number variation data |  | DepMap v21Q1 | https://depmap.org/portal/download/ | [92] |
| Promoter methylation data |  | CCLE v2018/10/22 | https://depmap.org/portal/download/ | [36] |
| Histone marks data |  | CCLE v2018/11/30 | https://depmap.org/portal/download/ | [36] |
| miRNA expression |  | CCLE v2018/11/03 | https://depmap.org/portal/download/ | [36] |
| Metabolite levels |  | CCLE v2019/05/02 | https://depmap.org/portal/download/ | [38] |
| Drug cell viability assays |  | PRISM v19Q4 | https://depmap.org/portal/download/ | [91] |
| CRISPR cell fitness data |  | Project Achilles v21Q1 | https://depmap.org/portal/download/ | [36] |
| Protein levels |  | CCLE v2020/01 | https://gygi.hms.harvard.edu/publications/ccle.html | [37] |

**Table S4 –** Experimental design and statistical methods for the analyses presented in the main text.

| Method | Analysis | Source | Link | Reference |
| --- | --- | --- | --- | --- |
| FIMO | Motif network inference | MEME suite v5.5.0 | https://meme-suite.org/meme/doc/fimo.html | [15] |
| PANDA | Gene regulatory network inference | netZooPy v0.8.1 | https://github.com/netZoo/netZooPy | [14] |
| LIONESS | Single-sample gene regulatory network reconstruction |  | https://github.com/netZoo/netZooPy | [20] |
| MONSTER | Estimation of regulatory drivers of transition between networks |  | https://github.com/netZoo/netZooPy | [28] |
| ANOVA | Assessing significance of association between TF targeting and various CCLE omics. | Statsmodels v0.13.2 | https://www.statsmodels.org/stable/index.html | [96] |
| Elastic net | Identifying TF targeting predictors of drug efficacy using | Sklearn v1.1.3 | https://scikit-learn.org/stable/ | [54] |
| DRAGON | Computing partial correlations between TF targeting and various omics layers. | netZooPy v0.8.1 | https://github.com/netZoo/netZooPy | [21] |

**Table S5** – Genes names of 1,132 TFs modeled in CCLE GRNs.

| **ID** | **TF** |
| --- | --- |
| 1 | AHR |
| 2 | AHRR |
| 3 | AIRE |
| 4 | ALX1 |
| 5 | ALX3 |
| 6 | ALX4 |
| 7 | ANHX |
| 8 | AR |
| 9 | ARGFX |
| 10 | ARID3A |
| 11 | ARID3B |
| 12 | ARID3C |
| 13 | ARID5A |
| 14 | ARID5B |
| 15 | ARNT |
| 16 | ARNT2 |
| 17 | ARNTL |
| 18 | ARNTL2 |
| 19 | ARX |
| 20 | ASCL1 |
| 21 | ASCL2 |
| 22 | ASCL3 |
| 23 | ASCL4 |
| 24 | ASCL5 |
| 25 | ATF1 |
| 26 | ATF2 |
| 27 | ATF3 |
| 28 | ATF4 |
| 29 | ATF5 |
| 30 | ATF6 |
| 31 | ATF6B |
| 32 | ATF7 |
| 33 | ATOH1 |
| 34 | ATOH7 |
| 35 | ATOH8 |
| 36 | BACH1 |
| 37 | BACH2 |
| 38 | BARHL1 |
| 39 | BARHL2 |
| 40 | BARX1 |
| 41 | BARX2 |
| 42 | BATF |
| 43 | BATF3 |
| 44 | BBX |
| 45 | BCL11A |
| 46 | BCL11B |
| 47 | BCL6 |
| 48 | BCL6B |
| 49 | BHLHA15 |
| 50 | BHLHE22 |
| 51 | BHLHE23 |
| 52 | BHLHE40 |
| 53 | BHLHE41 |
| 54 | BPTF |
| 55 | BSX |
| 56 | CDC5L |
| 57 | CDX1 |
| 58 | CDX2 |
| 59 | CDX4 |
| 60 | CEBPA |
| 61 | CEBPB |
| 62 | CEBPD |
| 63 | CEBPE |
| 64 | CEBPG |
| 65 | CEBPZ |
| 66 | CENPB |
| 67 | CIC |
| 68 | CLOCK |
| 69 | CPEB1 |
| 70 | CREB1 |
| 71 | CREB3 |
| 72 | CREB3L1 |
| 73 | CREB3L2 |
| 74 | CREB3L4 |
| 75 | CREB5 |
| 76 | CREBL2 |
| 77 | CREBZF |
| 78 | CREM |
| 79 | CRX |
| 80 | CTCF |
| 81 | CTCFL |
| 82 | CUX1 |
| 83 | CUX2 |
| 84 | CXXC1 |
| 85 | DBP |
| 86 | DBX1 |
| 87 | DBX2 |
| 88 | DDIT3 |
| 89 | DLX1 |
| 90 | DLX2 |
| 91 | DLX3 |
| 92 | DLX4 |
| 93 | DLX5 |
| 94 | DLX6 |
| 95 | DMBX1 |
| 96 | DMRT1 |
| 97 | DMRT2 |
| 98 | DMRT3 |
| 99 | DMRTA1 |
| 100 | DMRTA2 |
| 101 | DMRTC2 |
| 102 | DNMT1 |
| 103 | DPF1 |
| 104 | DPF3 |
| 105 | DPRX |
| 106 | DRGX |
| 107 | DUX4 |
| 108 | DUXA |
| 109 | E2F1 |
| 110 | E2F2 |
| 111 | E2F3 |
| 112 | E2F4 |
| 113 | E2F5 |
| 114 | E2F6 |
| 115 | E2F7 |
| 116 | E2F8 |
| 117 | E4F1 |
| 118 | EBF1 |
| 119 | EBF2 |
| 120 | EBF3 |
| 121 | EBF4 |
| 122 | EGR1 |
| 123 | EGR2 |
| 124 | EGR3 |
| 125 | EGR4 |
| 126 | EHF |
| 127 | ELF1 |
| 128 | ELF2 |
| 129 | ELF3 |
| 130 | ELF4 |
| 131 | ELF5 |
| 132 | ELK1 |
| 133 | ELK3 |
| 134 | ELK4 |
| 135 | EMX1 |
| 136 | EMX2 |
| 137 | EN1 |
| 138 | EN2 |
| 139 | EOMES |
| 140 | EPAS1 |
| 141 | ERF |
| 142 | ERG |
| 143 | ESR1 |
| 144 | ESR2 |
| 145 | ESRRA |
| 146 | ESRRB |
| 147 | ESRRG |
| 148 | ESX1 |
| 149 | ETS1 |
| 150 | ETS2 |
| 151 | ETV1 |
| 152 | ETV2 |
| 153 | ETV3 |
| 154 | ETV3L |
| 155 | ETV4 |
| 156 | ETV5 |
| 157 | ETV6 |
| 158 | ETV7 |
| 159 | EVX1 |
| 160 | EVX2 |
| 161 | FERD3L |
| 162 | FEV |
| 163 | FEZF1 |
| 164 | FEZF2 |
| 165 | FIGLA |
| 166 | FLI1 |
| 167 | FOS |
| 168 | FOSB |
| 169 | FOSL1 |
| 170 | FOSL2 |
| 171 | FOXA1 |
| 172 | FOXA2 |
| 173 | FOXA3 |
| 174 | FOXB1 |
| 175 | FOXB2 |
| 176 | FOXC1 |
| 177 | FOXC2 |
| 178 | FOXD1 |
| 179 | FOXD2 |
| 180 | FOXD3 |
| 181 | FOXD4 |
| 182 | FOXD4L1 |
| 183 | FOXD4L3 |
| 184 | FOXD4L4 |
| 185 | FOXD4L5 |
| 186 | FOXD4L6 |
| 187 | FOXE1 |
| 188 | FOXE3 |
| 189 | FOXF1 |
| 190 | FOXF2 |
| 191 | FOXG1 |
| 192 | FOXH1 |
| 193 | FOXI1 |
| 194 | FOXI2 |
| 195 | FOXI3 |
| 196 | FOXJ1 |
| 197 | FOXJ2 |
| 198 | FOXJ3 |
| 199 | FOXK1 |
| 200 | FOXK2 |
| 201 | FOXL1 |
| 202 | FOXL2 |
| 203 | FOXM1 |
| 204 | FOXN1 |
| 205 | FOXN2 |
| 206 | FOXN3 |
| 207 | FOXN4 |
| 208 | FOXO1 |
| 209 | FOXO3 |
| 210 | FOXO4 |
| 211 | FOXO6 |
| 212 | FOXP1 |
| 213 | FOXP2 |
| 214 | FOXP3 |
| 215 | FOXP4 |
| 216 | FOXQ1 |
| 217 | FOXR1 |
| 218 | FOXR2 |
| 219 | FOXS1 |
| 220 | GABPA |
| 221 | GATA1 |
| 222 | GATA2 |
| 223 | GATA3 |
| 224 | GATA4 |
| 225 | GATA5 |
| 226 | GATA6 |
| 227 | GBX1 |
| 228 | GBX2 |
| 229 | GCM1 |
| 230 | GCM2 |
| 231 | GFI1 |
| 232 | GFI1B |
| 233 | GLI1 |
| 234 | GLI2 |
| 235 | GLI3 |
| 236 | GLI4 |
| 237 | GLIS1 |
| 238 | GLIS2 |
| 239 | GLIS3 |
| 240 | GMEB1 |
| 241 | GMEB2 |
| 242 | GRHL1 |
| 243 | GRHL2 |
| 244 | GSC |
| 245 | GSC2 |
| 246 | GSX1 |
| 247 | GSX2 |
| 248 | GTF3A |
| 249 | HAND1 |
| 250 | HAND2 |
| 251 | HBP1 |
| 252 | HDX |
| 253 | HELT |
| 254 | HES1 |
| 255 | HES2 |
| 256 | HES3 |
| 257 | HES4 |
| 258 | HES5 |
| 259 | HES6 |
| 260 | HES7 |
| 261 | HESX1 |
| 262 | HEY1 |
| 263 | HEY2 |
| 264 | HEYL |
| 265 | HHEX |
| 266 | HIC1 |
| 267 | HIC2 |
| 268 | HIF1A |
| 269 | HIF3A |
| 270 | HINFP |
| 271 | HIVEP1 |
| 272 | HIVEP2 |
| 273 | HIVEP3 |
| 274 | HKR1 |
| 275 | HLF |
| 276 | HLX |
| 277 | HMBOX1 |
| 278 | HMG20B |
| 279 | HMGA1 |
| 280 | HMGA2 |
| 281 | HMX1 |
| 282 | HMX2 |
| 283 | HMX3 |
| 284 | HNF1A |
| 285 | HNF1B |
| 286 | HNF4A |
| 287 | HNF4G |
| 288 | HOMEZ |
| 289 | HOXA1 |
| 290 | HOXA10 |
| 291 | HOXA11 |
| 292 | HOXA13 |
| 293 | HOXA2 |
| 294 | HOXA3 |
| 295 | HOXA4 |
| 296 | HOXA5 |
| 297 | HOXA6 |
| 298 | HOXA7 |
| 299 | HOXA9 |
| 300 | HOXB1 |
| 301 | HOXB13 |
| 302 | HOXB2 |
| 303 | HOXB3 |
| 304 | HOXB4 |
| 305 | HOXB5 |
| 306 | HOXB6 |
| 307 | HOXB7 |
| 308 | HOXB8 |
| 309 | HOXB9 |
| 310 | HOXC10 |
| 311 | HOXC11 |
| 312 | HOXC12 |
| 313 | HOXC13 |
| 314 | HOXC4 |
| 315 | HOXC5 |
| 316 | HOXC6 |
| 317 | HOXC8 |
| 318 | HOXC9 |
| 319 | HOXD1 |
| 320 | HOXD10 |
| 321 | HOXD11 |
| 322 | HOXD12 |
| 323 | HOXD13 |
| 324 | HOXD3 |
| 325 | HOXD4 |
| 326 | HOXD8 |
| 327 | HOXD9 |
| 328 | HSF1 |
| 329 | HSF2 |
| 330 | HSF4 |
| 331 | HSF5 |
| 332 | IKZF1 |
| 333 | IKZF2 |
| 334 | IKZF3 |
| 335 | IKZF4 |
| 336 | INSM1 |
| 337 | IRF1 |
| 338 | IRF2 |
| 339 | IRF3 |
| 340 | IRF4 |
| 341 | IRF5 |
| 342 | IRF6 |
| 343 | IRF7 |
| 344 | IRF8 |
| 345 | IRF9 |
| 346 | IRX1 |
| 347 | IRX2 |
| 348 | IRX3 |
| 349 | IRX4 |
| 350 | IRX5 |
| 351 | IRX6 |
| 352 | ISL1 |
| 353 | ISL2 |
| 354 | ISX |
| 355 | JDP2 |
| 356 | JUN |
| 357 | JUNB |
| 358 | JUND |
| 359 | KDM2B |
| 360 | KLF1 |
| 361 | KLF10 |
| 362 | KLF11 |
| 363 | KLF12 |
| 364 | KLF13 |
| 365 | KLF14 |
| 366 | KLF15 |
| 367 | KLF16 |
| 368 | KLF17 |
| 369 | KLF2 |
| 370 | KLF3 |
| 371 | KLF4 |
| 372 | KLF5 |
| 373 | KLF6 |
| 374 | KLF7 |
| 375 | KLF8 |
| 376 | KLF9 |
| 377 | KMT2A |
| 378 | LBX1 |
| 379 | LBX2 |
| 380 | LCOR |
| 381 | LEF1 |
| 382 | LHX1 |
| 383 | LHX2 |
| 384 | LHX3 |
| 385 | LHX4 |
| 386 | LHX5 |
| 387 | LHX6 |
| 388 | LHX8 |
| 389 | LHX9 |
| 390 | LIN28A |
| 391 | LIN28B |
| 392 | LIN54 |
| 393 | LMX1A |
| 394 | LMX1B |
| 395 | LYL1 |
| 396 | MAF |
| 397 | MAFA |
| 398 | MAFB |
| 399 | MAFF |
| 400 | MAFG |
| 401 | MAFK |
| 402 | MAX |
| 403 | MAZ |
| 404 | MBD2 |
| 405 | MBNL2 |
| 406 | MECOM |
| 407 | MECP2 |
| 408 | MEF2A |
| 409 | MEF2B |
| 410 | MEF2C |
| 411 | MEF2D |
| 412 | MEIS1 |
| 413 | MEIS2 |
| 414 | MEIS3 |
| 415 | MEOX1 |
| 416 | MEOX2 |
| 417 | MESP1 |
| 418 | MESP2 |
| 419 | MGA |
| 420 | MITF |
| 421 | MIXL1 |
| 422 | MLX |
| 423 | MLXIP |
| 424 | MLXIPL |
| 425 | MNT |
| 426 | MNX1 |
| 427 | MSC |
| 428 | MSGN1 |
| 429 | MSX1 |
| 430 | MSX2 |
| 431 | MTF1 |
| 432 | MXD1 |
| 433 | MXD3 |
| 434 | MXD4 |
| 435 | MXI1 |
| 436 | MYB |
| 437 | MYBL1 |
| 438 | MYBL2 |
| 439 | MYC |
| 440 | MYCL |
| 441 | MYCN |
| 442 | MYF5 |
| 443 | MYF6 |
| 444 | MYNN |
| 445 | MYOD1 |
| 446 | MYOG |
| 447 | MYRF |
| 448 | MYT1L |
| 449 | MZF1 |
| 450 | NAIF1 |
| 451 | NANOG |
| 452 | NEUROD1 |
| 453 | NEUROD2 |
| 454 | NEUROD4 |
| 455 | NEUROD6 |
| 456 | NEUROG1 |
| 457 | NEUROG2 |
| 458 | NEUROG3 |
| 459 | NFAT5 |
| 460 | NFATC1 |
| 461 | NFATC2 |
| 462 | NFATC3 |
| 463 | NFATC4 |
| 464 | NFE2 |
| 465 | NFE2L1 |
| 466 | NFE2L2 |
| 467 | NFE2L3 |
| 468 | NFIA |
| 469 | NFIB |
| 470 | NFIC |
| 471 | NFIL3 |
| 472 | NFIX |
| 473 | NFKB1 |
| 474 | NFKB2 |
| 475 | NFYA |
| 476 | NFYB |
| 477 | NFYC |
| 478 | NHLH1 |
| 479 | NHLH2 |
| 480 | NKX1-1 |
| 481 | NKX1-2 |
| 482 | NKX2-1 |
| 483 | NKX2-2 |
| 484 | NKX2-3 |
| 485 | NKX2-4 |
| 486 | NKX2-5 |
| 487 | NKX2-6 |
| 488 | NKX2-8 |
| 489 | NKX3-1 |
| 490 | NKX3-2 |
| 491 | NKX6-1 |
| 492 | NKX6-2 |
| 493 | NKX6-3 |
| 494 | NOBOX |
| 495 | NOTO |
| 496 | NPAS1 |
| 497 | NPAS2 |
| 498 | NPAS3 |
| 499 | NR0B1 |
| 500 | NR1D1 |
| 501 | NR1D2 |
| 502 | NR1H2 |
| 503 | NR1H3 |
| 504 | NR1H4 |
| 505 | NR1I2 |
| 506 | NR1I3 |
| 507 | NR2C1 |
| 508 | NR2C2 |
| 509 | NR2E1 |
| 510 | NR2E3 |
| 511 | NR2F1 |
| 512 | NR2F2 |
| 513 | NR2F6 |
| 514 | NR3C1 |
| 515 | NR3C2 |
| 516 | NR4A1 |
| 517 | NR4A2 |
| 518 | NR4A3 |
| 519 | NR5A1 |
| 520 | NR5A2 |
| 521 | NR6A1 |
| 522 | NRF1 |
| 523 | NRL |
| 524 | OLIG1 |
| 525 | OLIG2 |
| 526 | OLIG3 |
| 527 | ONECUT1 |
| 528 | ONECUT2 |
| 529 | ONECUT3 |
| 530 | OSR1 |
| 531 | OSR2 |
| 532 | OTP |
| 533 | OTX1 |
| 534 | OTX2 |
| 535 | OVOL1 |
| 536 | OVOL2 |
| 537 | PATZ1 |
| 538 | PAX1 |
| 539 | PAX2 |
| 540 | PAX3 |
| 541 | PAX4 |
| 542 | PAX5 |
| 543 | PAX6 |
| 544 | PAX7 |
| 545 | PAX8 |
| 546 | PAX9 |
| 547 | PBX1 |
| 548 | PBX2 |
| 549 | PBX3 |
| 550 | PBX4 |
| 551 | PDX1 |
| 552 | PGR |
| 553 | PHOX2A |
| 554 | PHOX2B |
| 555 | PITX1 |
| 556 | PITX2 |
| 557 | PITX3 |
| 558 | PKNOX1 |
| 559 | PKNOX2 |
| 560 | PLAG1 |
| 561 | PLAGL1 |
| 562 | POU1F1 |
| 563 | POU2F1 |
| 564 | POU2F2 |
| 565 | POU2F3 |
| 566 | POU3F1 |
| 567 | POU3F2 |
| 568 | POU3F3 |
| 569 | POU3F4 |
| 570 | POU4F1 |
| 571 | POU4F2 |
| 572 | POU4F3 |
| 573 | POU5F1 |
| 574 | POU5F1B |
| 575 | POU6F1 |
| 576 | POU6F2 |
| 577 | PPARA |
| 578 | PPARD |
| 579 | PPARG |
| 580 | PRDM1 |
| 581 | PRDM14 |
| 582 | PRDM16 |
| 583 | PRDM4 |
| 584 | PRDM6 |
| 585 | PRDM9 |
| 586 | PROP1 |
| 587 | PROX1 |
| 588 | PROX2 |
| 589 | PRRX1 |
| 590 | PRRX2 |
| 591 | PTF1A |
| 592 | PURA |
| 593 | RARA |
| 594 | RARB |
| 595 | RARG |
| 596 | RAX |
| 597 | RAX2 |
| 598 | RBAK |
| 599 | RBPJ |
| 600 | RBPJL |
| 601 | REL |
| 602 | RELA |
| 603 | RELB |
| 604 | REST |
| 605 | RFX2 |
| 606 | RFX3 |
| 607 | RFX4 |
| 608 | RFX5 |
| 609 | RFX6 |
| 610 | RFX7 |
| 611 | RHOXF1 |
| 612 | RHOXF2 |
| 613 | RHOXF2B |
| 614 | RORA |
| 615 | RORB |
| 616 | RORC |
| 617 | RREB1 |
| 618 | RUNX1 |
| 619 | RUNX2 |
| 620 | RUNX3 |
| 621 | RXRA |
| 622 | RXRB |
| 623 | RXRG |
| 624 | SCRT1 |
| 625 | SCRT2 |
| 626 | SCX |
| 627 | SEBOX |
| 628 | SHOX |
| 629 | SHOX2 |
| 630 | SIM1 |
| 631 | SIM2 |
| 632 | SIX1 |
| 633 | SIX2 |
| 634 | SIX3 |
| 635 | SIX4 |
| 636 | SIX5 |
| 637 | SIX6 |
| 638 | SKOR1 |
| 639 | SKOR2 |
| 640 | SMAD1 |
| 641 | SMAD3 |
| 642 | SMAD4 |
| 643 | SMAD5 |
| 644 | SMAD9 |
| 645 | SNAI1 |
| 646 | SNAI2 |
| 647 | SNAI3 |
| 648 | SOHLH2 |
| 649 | SOX1 |
| 650 | SOX10 |
| 651 | SOX11 |
| 652 | SOX12 |
| 653 | SOX13 |
| 654 | SOX14 |
| 655 | SOX15 |
| 656 | SOX17 |
| 657 | SOX18 |
| 658 | SOX2 |
| 659 | SOX21 |
| 660 | SOX3 |
| 661 | SOX30 |
| 662 | SOX4 |
| 663 | SOX5 |
| 664 | SOX6 |
| 665 | SOX7 |
| 666 | SOX8 |
| 667 | SOX9 |
| 668 | SP1 |
| 669 | SP2 |
| 670 | SP3 |
| 671 | SP4 |
| 672 | SP5 |
| 673 | SP6 |
| 674 | SP7 |
| 675 | SP8 |
| 676 | SP9 |
| 677 | SPDEF |
| 678 | SPI1 |
| 679 | SPIB |
| 680 | SPIC |
| 681 | SPZ1 |
| 682 | SREBF1 |
| 683 | SREBF2 |
| 684 | SRF |
| 685 | ST18 |
| 686 | STAT1 |
| 687 | STAT2 |
| 688 | STAT3 |
| 689 | STAT4 |
| 690 | STAT5A |
| 691 | STAT5B |
| 692 | STAT6 |
| 693 | T |
| 694 | TAL1 |
| 695 | TAL2 |
| 696 | TBP |
| 697 | TBPL2 |
| 698 | TBR1 |
| 699 | TBX1 |
| 700 | TBX10 |
| 701 | TBX15 |
| 702 | TBX18 |
| 703 | TBX19 |
| 704 | TBX2 |
| 705 | TBX20 |
| 706 | TBX21 |
| 707 | TBX22 |
| 708 | TBX3 |
| 709 | TBX4 |
| 710 | TBX5 |
| 711 | TBX6 |
| 712 | TCF12 |
| 713 | TCF15 |
| 714 | TCF21 |
| 715 | TCF23 |
| 716 | TCF24 |
| 717 | TCF3 |
| 718 | TCF4 |
| 719 | TCF7 |
| 720 | TCF7L1 |
| 721 | TCF7L2 |
| 722 | TCFL5 |
| 723 | TEAD1 |
| 724 | TEAD2 |
| 725 | TEAD3 |
| 726 | TEAD4 |
| 727 | TEF |
| 728 | TERF2 |
| 729 | TET1 |
| 730 | TFAP2A |
| 731 | TFAP2B |
| 732 | TFAP2C |
| 733 | TFAP2D |
| 734 | TFAP2E |
| 735 | TFAP4 |
| 736 | TFCP2 |
| 737 | TFCP2L1 |
| 738 | TFDP1 |
| 739 | TFDP3 |
| 740 | TFE3 |
| 741 | TFEB |
| 742 | TFEC |
| 743 | TGIF1 |
| 744 | TGIF2 |
| 745 | TGIF2LX |
| 746 | THAP1 |
| 747 | THRA |
| 748 | THRB |
| 749 | TLX1 |
| 750 | TLX2 |
| 751 | TLX3 |
| 752 | TOPORS |
| 753 | TP53 |
| 754 | TP63 |
| 755 | TP73 |
| 756 | TWIST1 |
| 757 | TWIST2 |
| 758 | UBP1 |
| 759 | UNCX |
| 760 | USF1 |
| 761 | USF2 |
| 762 | VAX1 |
| 763 | VAX2 |
| 764 | VDR |
| 765 | VENTX |
| 766 | VSX1 |
| 767 | VSX2 |
| 768 | WT1 |
| 769 | XBP1 |
| 770 | XPA |
| 771 | YBX1 |
| 772 | YBX2 |
| 773 | YBX3 |
| 774 | YY1 |
| 775 | YY2 |
| 776 | ZBED1 |
| 777 | ZBTB1 |
| 778 | ZBTB12 |
| 779 | ZBTB14 |
| 780 | ZBTB18 |
| 781 | ZBTB2 |
| 782 | ZBTB20 |
| 783 | ZBTB22 |
| 784 | ZBTB26 |
| 785 | ZBTB3 |
| 786 | ZBTB32 |
| 787 | ZBTB33 |
| 788 | ZBTB34 |
| 789 | ZBTB37 |
| 790 | ZBTB4 |
| 791 | ZBTB42 |
| 792 | ZBTB43 |
| 793 | ZBTB45 |
| 794 | ZBTB48 |
| 795 | ZBTB49 |
| 796 | ZBTB6 |
| 797 | ZBTB7A |
| 798 | ZBTB7B |
| 799 | ZBTB7C |
| 800 | ZEB1 |
| 801 | ZEB2 |
| 802 | ZFHX2 |
| 803 | ZFHX3 |
| 804 | ZFHX4 |
| 805 | ZFP1 |
| 806 | ZFP14 |
| 807 | ZFP2 |
| 808 | ZFP28 |
| 809 | ZFP3 |
| 810 | ZFP30 |
| 811 | ZFP42 |
| 812 | ZFP57 |
| 813 | ZFP64 |
| 814 | ZFP69 |
| 815 | ZFP69B |
| 816 | ZFP82 |
| 817 | ZFP90 |
| 818 | ZFX |
| 819 | ZHX1 |
| 820 | ZIC1 |
| 821 | ZIC2 |
| 822 | ZIC3 |
| 823 | ZIC4 |
| 824 | ZIC5 |
| 825 | ZIK1 |
| 826 | ZIM2 |
| 827 | ZIM3 |
| 828 | ZKSCAN1 |
| 829 | ZKSCAN2 |
| 830 | ZKSCAN3 |
| 831 | ZKSCAN5 |
| 832 | ZKSCAN7 |
| 833 | ZNF10 |
| 834 | ZNF100 |
| 835 | ZNF101 |
| 836 | ZNF114 |
| 837 | ZNF12 |
| 838 | ZNF121 |
| 839 | ZNF124 |
| 840 | ZNF132 |
| 841 | ZNF133 |
| 842 | ZNF134 |
| 843 | ZNF135 |
| 844 | ZNF136 |
| 845 | ZNF140 |
| 846 | ZNF141 |
| 847 | ZNF143 |
| 848 | ZNF146 |
| 849 | ZNF148 |
| 850 | ZNF154 |
| 851 | ZNF157 |
| 852 | ZNF16 |
| 853 | ZNF169 |
| 854 | ZNF17 |
| 855 | ZNF174 |
| 856 | ZNF175 |
| 857 | ZNF177 |
| 858 | ZNF18 |
| 859 | ZNF180 |
| 860 | ZNF181 |
| 861 | ZNF182 |
| 862 | ZNF184 |
| 863 | ZNF189 |
| 864 | ZNF19 |
| 865 | ZNF197 |
| 866 | ZNF2 |
| 867 | ZNF200 |
| 868 | ZNF202 |
| 869 | ZNF205 |
| 870 | ZNF211 |
| 871 | ZNF212 |
| 872 | ZNF213 |
| 873 | ZNF214 |
| 874 | ZNF219 |
| 875 | ZNF22 |
| 876 | ZNF222 |
| 877 | ZNF223 |
| 878 | ZNF224 |
| 879 | ZNF225 |
| 880 | ZNF232 |
| 881 | ZNF235 |
| 882 | ZNF248 |
| 883 | ZNF25 |
| 884 | ZNF250 |
| 885 | ZNF254 |
| 886 | ZNF257 |
| 887 | ZNF26 |
| 888 | ZNF260 |
| 889 | ZNF263 |
| 890 | ZNF264 |
| 891 | ZNF266 |
| 892 | ZNF267 |
| 893 | ZNF273 |
| 894 | ZNF274 |
| 895 | ZNF276 |
| 896 | ZNF28 |
| 897 | ZNF280A |
| 898 | ZNF281 |
| 899 | ZNF282 |
| 900 | ZNF283 |
| 901 | ZNF284 |
| 902 | ZNF285 |
| 903 | ZNF287 |
| 904 | ZNF296 |
| 905 | ZNF3 |
| 906 | ZNF30 |
| 907 | ZNF300 |
| 908 | ZNF302 |
| 909 | ZNF304 |
| 910 | ZNF311 |
| 911 | ZNF317 |
| 912 | ZNF32 |
| 913 | ZNF320 |
| 914 | ZNF322 |
| 915 | ZNF324 |
| 916 | ZNF324B |
| 917 | ZNF329 |
| 918 | ZNF331 |
| 919 | ZNF333 |
| 920 | ZNF334 |
| 921 | ZNF337 |
| 922 | ZNF33A |
| 923 | ZNF33B |
| 924 | ZNF34 |
| 925 | ZNF341 |
| 926 | ZNF343 |
| 927 | ZNF345 |
| 928 | ZNF35 |
| 929 | ZNF350 |
| 930 | ZNF354A |
| 931 | ZNF354B |
| 932 | ZNF354C |
| 933 | ZNF37A |
| 934 | ZNF382 |
| 935 | ZNF383 |
| 936 | ZNF384 |
| 937 | ZNF385D |
| 938 | ZNF394 |
| 939 | ZNF396 |
| 940 | ZNF398 |
| 941 | ZNF41 |
| 942 | ZNF410 |
| 943 | ZNF415 |
| 944 | ZNF416 |
| 945 | ZNF417 |
| 946 | ZNF418 |
| 947 | ZNF419 |
| 948 | ZNF423 |
| 949 | ZNF425 |
| 950 | ZNF429 |
| 951 | ZNF430 |
| 952 | ZNF431 |
| 953 | ZNF432 |
| 954 | ZNF433 |
| 955 | ZNF436 |
| 956 | ZNF439 |
| 957 | ZNF44 |
| 958 | ZNF440 |
| 959 | ZNF441 |
| 960 | ZNF442 |
| 961 | ZNF443 |
| 962 | ZNF444 |
| 963 | ZNF445 |
| 964 | ZNF449 |
| 965 | ZNF45 |
| 966 | ZNF454 |
| 967 | ZNF460 |
| 968 | ZNF467 |
| 969 | ZNF468 |
| 970 | ZNF479 |
| 971 | ZNF480 |
| 972 | ZNF483 |
| 973 | ZNF484 |
| 974 | ZNF485 |
| 975 | ZNF486 |
| 976 | ZNF487 |
| 977 | ZNF490 |
| 978 | ZNF492 |
| 979 | ZNF496 |
| 980 | ZNF501 |
| 981 | ZNF502 |
| 982 | ZNF506 |
| 983 | ZNF513 |
| 984 | ZNF519 |
| 985 | ZNF524 |
| 986 | ZNF525 |
| 987 | ZNF527 |
| 988 | ZNF528 |
| 989 | ZNF529 |
| 990 | ZNF530 |
| 991 | ZNF534 |
| 992 | ZNF540 |
| 993 | ZNF543 |
| 994 | ZNF547 |
| 995 | ZNF548 |
| 996 | ZNF549 |
| 997 | ZNF550 |
| 998 | ZNF552 |
| 999 | ZNF554 |
| 1000 | ZNF555 |
| 1001 | ZNF557 |
| 1002 | ZNF558 |
| 1003 | ZNF561 |
| 1004 | ZNF562 |
| 1005 | ZNF563 |
| 1006 | ZNF564 |
| 1007 | ZNF565 |
| 1008 | ZNF566 |
| 1009 | ZNF567 |
| 1010 | ZNF570 |
| 1011 | ZNF571 |
| 1012 | ZNF573 |
| 1013 | ZNF574 |
| 1014 | ZNF580 |
| 1015 | ZNF582 |
| 1016 | ZNF584 |
| 1017 | ZNF585A |
| 1018 | ZNF586 |
| 1019 | ZNF587 |
| 1020 | ZNF589 |
| 1021 | ZNF594 |
| 1022 | ZNF595 |
| 1023 | ZNF596 |
| 1024 | ZNF597 |
| 1025 | ZNF605 |
| 1026 | ZNF610 |
| 1027 | ZNF611 |
| 1028 | ZNF613 |
| 1029 | ZNF614 |
| 1030 | ZNF615 |
| 1031 | ZNF616 |
| 1032 | ZNF619 |
| 1033 | ZNF620 |
| 1034 | ZNF621 |
| 1035 | ZNF626 |
| 1036 | ZNF627 |
| 1037 | ZNF641 |
| 1038 | ZNF649 |
| 1039 | ZNF653 |
| 1040 | ZNF655 |
| 1041 | ZNF660 |
| 1042 | ZNF662 |
| 1043 | ZNF667 |
| 1044 | ZNF669 |
| 1045 | ZNF671 |
| 1046 | ZNF674 |
| 1047 | ZNF675 |
| 1048 | ZNF677 |
| 1049 | ZNF680 |
| 1050 | ZNF681 |
| 1051 | ZNF682 |
| 1052 | ZNF684 |
| 1053 | ZNF69 |
| 1054 | ZNF691 |
| 1055 | ZNF692 |
| 1056 | ZNF695 |
| 1057 | ZNF7 |
| 1058 | ZNF701 |
| 1059 | ZNF704 |
| 1060 | ZNF705G |
| 1061 | ZNF707 |
| 1062 | ZNF708 |
| 1063 | ZNF71 |
| 1064 | ZNF711 |
| 1065 | ZNF713 |
| 1066 | ZNF714 |
| 1067 | ZNF716 |
| 1068 | ZNF730 |
| 1069 | ZNF736 |
| 1070 | ZNF737 |
| 1071 | ZNF74 |
| 1072 | ZNF740 |
| 1073 | ZNF749 |
| 1074 | ZNF75A |
| 1075 | ZNF75D |
| 1076 | ZNF76 |
| 1077 | ZNF764 |
| 1078 | ZNF765 |
| 1079 | ZNF766 |
| 1080 | ZNF768 |
| 1081 | ZNF77 |
| 1082 | ZNF770 |
| 1083 | ZNF771 |
| 1084 | ZNF774 |
| 1085 | ZNF776 |
| 1086 | ZNF777 |
| 1087 | ZNF778 |
| 1088 | ZNF780A |
| 1089 | ZNF782 |
| 1090 | ZNF783 |
| 1091 | ZNF784 |
| 1092 | ZNF785 |
| 1093 | ZNF786 |
| 1094 | ZNF787 |
| 1095 | ZNF789 |
| 1096 | ZNF79 |
| 1097 | ZNF790 |
| 1098 | ZNF791 |
| 1099 | ZNF792 |
| 1100 | ZNF793 |
| 1101 | ZNF799 |
| 1102 | ZNF8 |
| 1103 | ZNF805 |
| 1104 | ZNF808 |
| 1105 | ZNF81 |
| 1106 | ZNF816 |
| 1107 | ZNF821 |
| 1108 | ZNF823 |
| 1109 | ZNF84 |
| 1110 | ZNF846 |
| 1111 | ZNF85 |
| 1112 | ZNF852 |
| 1113 | ZNF860 |
| 1114 | ZNF879 |
| 1115 | ZNF880 |
| 1116 | ZNF891 |
| 1117 | ZNF90 |
| 1118 | ZNF93 |
| 1119 | ZNF98 |
| 1120 | ZSCAN1 |
| 1121 | ZSCAN10 |
| 1122 | ZSCAN16 |
| 1123 | ZSCAN22 |
| 1124 | ZSCAN23 |
| 1125 | ZSCAN26 |
| 1126 | ZSCAN29 |
| 1127 | ZSCAN30 |
| 1128 | ZSCAN31 |
| 1129 | ZSCAN4 |
| 1130 | ZSCAN5A |
| 1131 | ZSCAN5C |
| 1132 | ZSCAN9 |
