# Supplementary material for: The Network Zoo: a multilingual package for the inference and analysis of gene regulatory networks
Source: Genome Biol. 2023 Mar 9;24:45. doi: 10.1186/s13059-023-02877-1 (PMC9999668; doi:10.1186/s13059-023-02877-1)
Supplement: Supplementary file 2 — Additional file 2. Review history. [file 13059_2023_2877_MOESM2_ESM.docx]

**1^st^ round**

**Reviewer 1**

===

This manuscript presents a software NetZoo for network inference and analysis. The software is implemented in multiple programming languages; netZooR for R, netZooPy for Python, netZooM for Matlab, and netZooC for C. Network inference and analysis have impact in broad field, and I believe netZoo will be a useful tool. I have one concerns about the software.

1. In total, 13 methods are implemented in netZoo; Those are PANDA, LIONESS, PUMA, CONDOR,

ALPACA, SAMBAR, SPIDER, MONSTER, OTTER, DRAGON, CRANE, EGRET, and YARN. netZooR implements 10 methods, netZooPy implements 7 methods, netZooM implements 5 methods, and netZooC implements 2 methods. I appreciate authors implement those methods in multiple languages so that user can choose one language they most familiar to use netZoo. However, none of the languages implements all 13 methods. This is not convenient for users since most users are familiar with only one language. I suggest authors to all 13 methods in either R or Python.

===

Please consider the following questions:

-Is the Software likely to be of broad utility? Is it easy to install and use?

-Does the software represent a significant advance over previously published tools (as demonstrated by direct comparison with available related software)?

-Please indicate briefly the novel features and/or advantages of the software and/or please reference the relevant publications and which alternatives, if any, it should be compared with.

-Are the methods appropriate to the aims of the study, are they well described, and are necessary controls included?

-Are the conclusions adequately supported by the data shown?

-Are sufficient details provided to allow replication and comparison with related analyses that may have been performed?

-Is the paper of broad interest to others in the field, or of outstanding interest to a broad audience of biologists?

**Reviewer 2**

The paper entitled "The Network Zoo: a multilingual package for the inference and analysis of biological networks" aims at describing a software tool gathering a wide range of methods dedicated to gene regulatory network inference and analysis. The work is of interest as the tools are used in the community, useful to extract knowledge from omics data, and complementary to each other.

Having these different tools in a common framework can hence be beneficial. However, the manuscript lacks clarity, focus, and consistency, making complicated a true understanding of the work that has been done and its potential impact/utility. For instance, the paper fails to clearly describe the work that has been conducted to harmonize the tools. And, importantly, the relationships of the work presented here with the companion tools and papers describing the produced networks (https://academic.oup.com/nar/article/50/D1/D610/6368528?login=false) and the notebooks associated with the methods (https://www.nature.com/articles/s41592-022-01479-2) are unclear.

General comments

- There is a lot of ambiguity and vagueness in the use of the words "integration" "model/modeling" "inference" "omics/multi-omics", which are used in a large variety of combinations. It is not clear if there are some nuances or if all combinations of words are synonymes. For instance, it seems that "network modeling" and "network inference" are used as synonyms; sometimes it's "multi-omics modeling" instead of network modeling. Other time it's "network estimation" or even "estimate network model"... Is there a difference in between all these different semantics? Between "model biological network" versus "inference of cellular biological networks"? The verb "to model" is used also for different purposes, e.g. to model networks or to model the transition between biological states. This is making the paper overall hard to follow.

- In the additional information form, "experimental design and statistics" as well as "Resources" are noted as "not applicable". I don't think this is the case as a wide range of resources, from existing tools to data, are used in netZoo. The manuscript should clarify the different resources that are used, maybe with a FAIRification process for the omics and knowledge datasets and the workflows. For instance, to reproduce the analyses presented in the manuscript, which data, stored where, should be used?

- In addition, the manuscript should be more clear with regard to its relationships to the two tightly linked manuscripts from the same team: the Jupyter notebooks, which intends to provide use-cases and illustrate the use of the methods, and GRAND, which intend to store output GRNs. The conclusion of the present manuscript, for instance, mentions that netZoo is doing all of these.

- The use-case based on LIONESS and PANDA is similar (from a protocol point of view) to the example presented in the LIONESS paper. The added value of netZoo is hence not really emphasized by this example.

Title and abstract

- the focus of the work could be more clear. In particular, "biological networks" is used in the title and in other places, including in the abstract. The focus of the methods gathered here seems more dedicated to the inference of gene regulatory networks, which is not clear by reading the title and abstract. Biological networks could be understood as including protein-protein interaction networks or other types of networks. This should be clarified for the reader to know from the beginning what is the purpose of the netZoo software.

- multilingual is not straightforward neither. The fact that the different tools are in different langages is mentioned but the results and methods sections do not clearly explain how everything has been harmonized.

- tools/toolkit/software/software tools => switch singular/plural and between synonymes can blur the messages.

- "as additional data types become available", the data types that can be used by the different methods have not been clearly mentioned. It is hence difficult to get what additional data types could correspond to.

Background

- "Defining these interactions can be modeled by assessing..." unclear.

- "Each method is based on modeling known biological interactions" => unclear. To me, inferring interactions from omics data is different from modeling known biological interactions. And if the interactions are known, what is the purpose of modeling them (and what does it mean?)?

- "for estimation of multi-omic networks based on GGMs" => what is a multi-omics network, how a network can be "estimated", what is the difference between estimating, inferring or modeling a network? And what is a GGMs? It is the first mention of GGMs in the manuscript and it has not - to my knowledge - be defined anywhere.

- "TF-gene interactions In addition", missing dot.

- The description of the methods for GRN inference in the background section is brief, making it confusing. At the same time, the first section of the results is also dedicated to the description of the same list of methods (whereas these descriptions are more background than results). Maybe the paper should be reorganized, and the methods should be described only once in-depth.

- I don't get the reference to the "No free Lunch Theorem" to describe the fact that the different methods share a common framework.

- "centralized resource for biological networks" => isn't this more the goal of GRAND or NDEX? Furthermore, this sentence is unclear.

Results

- The paper lacks description on how the tools were harmonized and adapted to the different langages. This could be a first section, which would also include the description of the community ecosystem that is currently available after the description of the use-cases.

- The paper lacks description of the different omics datasets that can be used as input for the different methods. Different datasets are also mentioned without description. For instance "gene knockdown" or "fitness". The method section does not provide details neither.

- The method section is not referenced in the Result section. The result and method sections do not provide enough information and details to understand the experiment that have been conducted and the results obtained. The split of the text between method and result sections is sometimes not clear, with methodological details available from both places.

- "assessing significance of differential modules" unclear

- "TF targeting" has not been clearly defined. I'm not sure to understand what biological process it corresponds to, and what is the rationale for linking it with outgoing edge weights. Is "TF targeting" and "targetting by the TF" the same process?

-"we assume that if a gene product is repressed by miRNA, then it is likely nonessential": this sounds like a very strong statement, which - a minima - should be backed up by references.

- "with a context+ score of -0.16" this is unclear, it assumes the reader is very familiar with the methods and their outputs.

- "this suggests that these metabolites are upstream of LDHA" I don't get why.

- "this effect could be dominant in CCLE, since we did not filter ..." I don't get why.

- Are the ANOVA and ElasticNet approaches presented here implemented in netZoo?

-"we systematically overlaid networks based on our understanding of the cascade of regulatory processes": how? Manually?

Conclusion

- "multi-omics gene regulatory and partial correlation networks": the semantic used here is different from the one used in the abstract and introduction, not clear what method/approach it refers to.

- "in line with recent similar effort": the conclusion does not seem the be the correct place to mention existing effort and state of the art, that are in addition lacking in the background section

- "sharing of use cases", "continued development and maintenance", "community participation", these have not been described, to the best of my knowledge.

- "hosting networks" isn't this more the goal of GRAND than of netZoo?

Methods

- method section is not presented in the same order than the results

-"running FIMO": is FIMO also available in netZoo?

-"gene-gene correlations network based on expression", more details on how the correlations hare computed, from what type of data, would be pertinent.

-"using the mode parameter to intersection which take the intersection" not clear

- ANOVA model: why discretizing the data before doing the ANOVA, and not using the raw data?

- "positive instance for methylated promoter/amplified genes and negative instances for nonmethylated promoters/nonamplified genes" this is counter intuitive to me as methylation inhibits genes expression, contrarily to gene amplification. What is the rationale for pairing opposite biological signals?

- Data used from CCLE are poorly described.

Figure 1

- arrows are not clear, for instance it seems that gene co-expression is used only by PANDA and not by OTTER.

- description of omics data used by the different methods could be improved.

- why are some tools overlapping (eg PANDA and OTTER) and not others?

- is the output of SAMBA linked to MONSTER?

- ALPACA/CRANE why two methods for one goal?

- Some methods are described in the caption but not in the figure.

- There are some discrepancies between the caption and the figure, in the description of the methods. For instance, MONSTER "estimates a transition matrix between two networks" or "estimates network transition drivers". This lead to unnecessary complexities.

Figure 4B and C, edge colors are not defined.

**Reviewer 3**

Inferring gene regulatory networks from collections of omics profile is a critical component in studying complex molecular phenomena underlying health and disease. Over the years, the Quackenbush has developed an incredible array of methods and software tools to reconstruct, compare, and contrast context-specific such networks. In this manuscript, they describe netZoo, a unified software framework that harmonizes the implementations of all these tools under one roof, enabling researchers to apply them in an integrative manner. With implementations in multiple programming languages, tools to ensure code consistency and robustness, and features for community support, netZoo is likely to have major impact.

At this point, though the achievements of this effort can be inferred, the manuscript is in serious need of some key details and descriptions.

Major comments/questions

=================

1. What did harmonizing the previous implementations of these tools entail despite them sharing common methodologies and computational cores? What were the challenges in this harmonization process in terms of the underlying analytical methods, data structures, numerical routines, memory/space constraints, etc.?

2. The authors lay out a detailed CCLE analysis. But, this whole description is not serving the main point this manuscript is hoping to make, which is: how did this extensive analysis become possible and/or easier with netZoo and why wasn't is possible to do exactly the same analysis just using the individual tools as they were available from their previous implementations? In the context of this manuscript, the authors need to rewrite to use these cool analyses as a backdrop to talk about the challenges in using existing implementations and what specific things they developed in netZoo that enabled them to overcome these challenges.

3. The development team has done a commendable job making many tools within netZoo available in multiple programming languages. However, why did they end up with different subsets of tools being implemented in R, Python, MATLAB, and C? The question is not to ask for all tools in all languages. Instead, the authors need to discuss this in the manuscript: how and why did the authors make these choices of tools for different languages.

Minor comments/questions

=================

1. The various tools — from PANDA to MONSTER — are described to different extents more than once in the manuscript. This is unnecessary. In fact, a single detailed description of each tool can be put together in one place and provided as part of the supplement, leaving just a high-level outline in the main text.

2. Page 9, end of the first paragraph under "Modeling TF targeting associations in melanoma": … if it had a z-score of less than *negative* three.

**Authors’ response**

**Reviewer #1:**

This manuscript presents a software NetZoo for network inference and analysis. The software is implemented in multiple programming languages; netZooR for R, netZooPy for Python, netZooM for Matlab, and netZooC for C. Network inference and analysis have impact in broad field, and I believe netZoo will be a useful tool. I have one concerns about the software.

1. In total, 13 methods are implemented in netZoo; Those are PANDA, LIONESS, PUMA, CONDOR, ALPACA, SAMBAR, SPIDER, MONSTER, OTTER, DRAGON, CRANE, EGRET, and YARN. netZooR implements 10 methods, netZooPy implements 7 methods, netZooM implements 5 methods, and netZooC implements 2 methods. I appreciate authors implement those methods in multiple languages so that user can choose one language they most familiar to use netZoo. However, none of the languages implements all 13 methods. This is not convenient for users since most users are familiar with only one language. I suggest authors to all 13 methods in either R or Python.

-> We thank the referee for their positive comments about NetZoo and his enthusiasm for the overall collection of methods. We have been working to implement all of the methods in both R and Python and as of this resubmission, all thirteen, including SPIDER, PUMA, and DRAGON, are now implemented in R and we hope to have broader representation of methods in Python in the near future.

**Reviewer #2:**

The paper entitled "The Network Zoo: a multilingual package for the inference and analysis of biological networks" aims at describing a software tool gathering a wide range of methods dedicated to gene regulatory network inference and analysis. The work is of interest as the tools are used in the community, useful to extract knowledge from omics data, and complementary to each other.

Having these different tools in a common framework can hence be beneficial. However, the manuscript lacks clarity, focus, and consistency, making complicated a true understanding of the work that has been done and its potential impact/utility. For instance, the paper fails to clearly describe the work that has been conducted to harmonize the tools. And, importantly, the relationships of the work presented here with the companion tools and papers describing the produced networks (<https://academic.oup.com/nar/article/50/D1/D610/6368528?login=false>) and the notebooks associated with the methods (<https://www.nature.com/articles/s41592-022-01479-2>) are unclear.

General comments

- There is a lot of ambiguity and vagueness in the use of the words "integration" "model/modeling" "inference" "omics/multi-omics", which are used in a large variety of combinations. It is not clear if there are some nuances or if all combinations of words are synonymes. For instance, it seems that "network modeling" and "network inference" are used as synonyms; sometimes it's "multi-omics modeling" instead of network modeling. Other time it's "network estimation" or even "estimate network model"... Is there a difference in between all these different semantics? Between "model biological network" versus "inference of cellular biological networks"? The verb "to model" is used also for different purposes, e.g. to model networks or to model the transition between biological states. This is making the paper overall hard to follow.

-> We thank the refeee for pointing out the potential confusion about the use of various terms. In our own work, we use these terms more of less interchangeably, but it recognize that for those not steeped in these methods, this can be confusing. For clarity we have chosen to use the phrase “network inference” instead of “network modeling/network estimation/estimate network model/model biological network” throughout

We also use the term “cellular network” to refer the physical network operating in the cell. that occur in the cell and distinguish from “ecological” networks that can be “biological” too, but we chose to use “gene regulatory networks” as opposed to biological networks as referred to in a previous comment. We removed the verb “to model” from any reference to network inference and used the word inference instead, and for “transition modeling” we replaced it with ”transition estimation”. We’ve also used the word ‘network’ instead of ‘model’.

We reserved the word “model” for “modeling of biological systems/gene regulatory processes”.

Throughout the text, “Omic” refers to a single measured biological variable (transcriptomics, proteomics, metabolomics for example), multi-omics refers to a combination of two or more of these omics such as the case for network that integrates mutli-omic data. We resolved the occurrences in the text where this has not been clear.

As for “integration”, it had only two occurrences where the primary meaning of the term was used, also, ‘continuous integration’ has been used in the text, which a specific technical term to refer to unit testing for software commonly referred to as continuous integration/continuous development (CI/CD)

- In the additional information form, "experimental design and statistics" as well as "Resources" are noted as "not applicable". I don't think this is the case as a wide range of resources, from existing tools to data, are used in netZoo. The manuscript should clarify the different resources that are used, maybe with a FAIRification process for the omics and knowledge datasets and the workflows. For instance, to reproduce the analyses presented in the manuscript, which data, stored where, should be used?

-> As a response to the referee’s excellent suggestion, we added a “resources” table (table S3) and “experimental design” table (table s4) in supplementary information file. We also added code to reproduce the analysis and added to our Jupyter Notebook server (netbooks.networkmedicine.org).

- In addition, the manuscript should be more clear with regard to its relationships to the two tightly linked manuscripts from the same team: the Jupyter notebooks, which intends to provide use-cases and illustrate the use of the methods, and GRAND, which intend to store output GRNs. The conclusion of the present manuscript, for instance, mentions that netZoo is doing all of these.

-> We thank the referee for giving us the opportunity to clarify this point. This manuscript presents our work on developing the method “codebase” which we refer to as the netZoo, a collection of methods in various programming languages hosted on GitHub. The “netZoo ecosystem”-which the reviewer is referring to and mentioned in the conclusion of the paper- has been extensively detailed in the results section under “Creating a community ecosystem for collaborative software development” and illustrated in Figure 5. Briefly, the netZoo ecosystem is the combination of the online tools (GRAND database, Jupyter Notebook web server and the netZoo codebase) that together support the process of biomedical investigation using gene regulatory network inference. In all our investigations that use the netZoo codebase, including this present work, we host the code on Netbooks and the networks on GRAND. This ecosystem of interoperable online tools makes it easier for developers to publish reproducible analyses and for users to access data and code for each analysis, without having to re-generate gigabytes of data. These tools are connected and will continue to grow together as they are updated and developed. For example, updating the netZoo codebase will update the Jupyter Notebook analysis in Netbooks, we are also working on re-generating networks in GRAND with each new netZoo release. To support this idea, we released a new major Netbooks version (v 2.0) with this current manuscript. This release includes new use cases and demonstrates the continued growth of these resources beyond what has been described in the original Netbooks publication. We clarified this further in the text in the final section of results

- The use-case based on LIONESS and PANDA is similar (from a protocol point of view) to the example presented in the LIONESS paper. The added value of netZoo is hence not really emphasized by this example.

-> We recognize that our description did not convey the novelty of this use case and we are sorry for the confusion that might have occurred. In the use case presented in this paper, we added a MONSTER network transition analysis on top of the PANDA-LIONESS inferred networks. This wouldn’t have been possible previously because we developed an interface within netZoo that links MONSTER with PANDA-LIONESS. The original implementation of MONSTER had an initial step that generated two gene regulatory networks using gene expression data and a second step that estimates transition between them. Reimplementing MONSTER in netZoo allowed the creation of a natural way to link the transition analysis to PANDA-LIONESS networks.

We also agree that from a protocol point of view, the pair PANDA and LIONESS has been employed for many years and for dozens of publications including the LIONESS paper.

The success of combining these two methods was our motivation in creating netZoo because we were interested in finding new synergies between methods that would ultimately give new biological insights. To do so, we chose CCLE data as our use case because despite having information about more than 7 omics, gene regulation was not characterized, which shows that computational modeling can be an alternative for the lack of large-scale experimental techniques.

CCLE data is the perfect example to show that netZoo is uniquely positioned to model complex multiomic data. In particular, CCLE has one single gene expression sample for each cell line, making it impossible for aggregate network methods (coexpression, ARACNE, WGCNA) to model them. However, LIONESS can model networks using a single gene expression sample using population data as a background. In contrast, the case presented in LIONESS paper had several replicates per sample, which was chosen specifically to show that LIONESS networks are reproducible between replicates. Therefore, these two dataset were carefully selected to serve different purposes in these 2 publications, each using a specific way of combining PANDA and LIONESS. We expanded on this further in the result section “Estimating TF targeting in melanoma CCLE cell lines”.

Title and abstract

- the focus of the work could be more clear. In particular, "biological networks" is used in the title and in other places, including in the abstract. The focus of the methods gathered here seems more dedicated to the inference of gene regulatory networks, which is not clear by reading the title and abstract. Biological networks could be understood as including protein-protein interaction networks or other types of networks. This should be clarified for the reader to know from the beginning what is the purpose of the netZoo software.

-> Thank you for this great suggestion, we agree with it and we changed it to gene regulatory networks.

- multilingual is not straightforward neither. The fact that the different tools are in different languages is mentioned but the results and methods sections do not clearly explain how everything has been harmonized.

-> Thank you for pointing out this shortcoming of our work. One aspect that makes netZoo truly a “multilingual” project is that we harmonized the implementation of various tools by comparing their outputs and resolving differences due to the programming language. This has helped us improve reproducibility, and develop guidelines for multilingual methods that were developed subsequently (such as OTTER and DRAGON). This includes the development of cross-platform unit tests that wouldn't be possible if methods are not hosted in a unique central resource. We have expanded on this concept in the first section of the results “netZoo integrates network inference and downstream analyses”.

- tools/toolkit/software/software tools => switch singular/plural and between synonymes can blur the messages.

-> As these synonyms might confuse the reader, we now use the word “methods” throughout the text. Also, we now reserve the word “tool” to refer to any component of the online netZoo ecosystem (GRAND database, Netbooks webserver) as opposed to individual methods (PANDA, LIONESS, DRAGON).

- "as additional data types become available", the data types that can be used by the different methods have not been clearly mentioned. It is hence difficult to get what additional data types could correspond to.

-> As a response to this comment and to clarify the abstract, we detailed the seven data modalities that we used in the CCLE analysis using netZoo in the abstract. These data correspond to mutation status, copy number variation, methylation status, gene and microRNA expression, protein levels, and metabolite concentrations. In addition, we associated them to cellular ‘phenotypic’ variables from CCLE such as drug viability and CRISPR knockout screens i.e., cell fitness. In the future, we will add methods to model networks in single-cell genomics assays and we added a mention of this perspective in the conclusion.

Background

- "Defining these interactions can be modeled by assessing..." unclear.

-> To clarify this sentence, we changed it to “These interactions can be modeled by assessing”

- "Each method is based on modeling known biological interactions" => unclear. To me, inferring interactions from omics data is different from modeling known biological interactions. And if the interactions are known, what is the purpose of modeling them (and what does it mean?)?

-> Thank you for giving us the opportunity to clarify this point. Our approach for biological network inference from omics data, uses prior knowledge (known interactions) to guide the learning process.

For example, if we know that a TF does not have a binding site in the promoter region of gene (known absence of interaction), and if transcriptomics tell us that this TF-gene pair are highly co-expressed, then we will identify this interaction as false positive, given the prior information we had. For example, PANDA downweighs network edges learned from gene expression if they’re not supported by TF motifs, and known PPI interactions. Both of these prior networks (PPI+coexpression) are provided as initial conditions for the inference on a given omics dataset.

This prior information can be generic for a species, or can be modeled for specific contexts, such as a cell line or a disease.

Several studies (including Culos and colleagues 2020, DOI: [10.5281/zenodo.3885868](https://doi.org/10.5281/zenodo.3885868).) have shown that including prior biological knowledge is an asset in network inference. We have also mentioned the “no-free lunch” theorem from optimization that formalizes this idea at a higher level, but this concept has been revisited in machine learning through the “learning using privileged information” paradigm (Vapnik and colleagues, 2015).

The natural theoretical framework for the idea of using prior knowledge is Bayesian statistics that conducts inference from the data using constraints from a known prior distribution, and we are currently developing more methods that use these concepts explicitly. We have clarified this point in the corresponding section of the text and also in the “no-free lunch theorem” question below.

- "for estimation of multi-omic networks based on GGMs" => what is a multi-omics network, how a network can be "estimated", what is the difference between estimating, inferring or modeling a network? And what is a GGMs? It is the first mention of GGMs in the manuscript and it has not - to my knowledge - be defined anywhere.

-> “Estimating”, “inferring“, and “modeling“ are synonyms that we used for variety to improve readability in the text but we also realize that it may confuse readers, therefore we chose “infer” to describe network inference and have removed “model” and “estimate”.

A GGM is Gaussian Graphical Model, a particular network inference technique that represents edge weights between nodes of the network as partial correlations, that is, correlations that are corrected for all other nodes in the network and thus may detect direct relationships, while Pearson’s correlation can have spurious correlations if intermediate variables mediate the effect between two nodes, including indirect effects, which is likely to occur in high-dimensional datasets. We have clarified the GGM idea in the text and and explained its abbreviation.

- "TF-gene interactions In addition", missing dot.

-> We added the missing dot in the text.

- The description of the methods for GRN inference in the background section is brief, making it confusing. At the same time, the first section of the results is also dedicated to the description of the same list of methods (whereas these descriptions are more background than results). Maybe the paper should be reorganized, and the methods should be described only once in-depth.

-> We removed the descriptions in the results, keeping only those in the background section and expanded on them. We also added detailed descriptions as a supplementary text and refer the reader to them.

- I don't get the reference to the "No free Lunch Theorem" to describe the fact that the different methods share a common framework.

-> The 1997 Wolpert and MacReady No Free Lunch Theorem (NFL) paper set the intellectual framework that we have used to guide the development of the complex optimization methods that are used for network inference. The conclusion of the NFL paper reads “These same results also indicate the importance of incorporating problem-specific knowledge into the behavior of the algorithm.” What we have found repeatedly is that using expression correlation or protein-protein interactions alone yield results that are unsatisfactory in that they lack true biological explanatory power for the systems we are studying, but that including prior knowledge of the likely TF-gene regulatory network and using other sources of data in a way that respects our knowledge of the interactions that are likely to occur, allow us to optimize around the initial network structure to converge on a meaningful final network. This was shown fairly convincingly in a recent analysis that compared two pancreatic cancer subtypes and showed how regulatory network inference provided deeper insight into disease processes than did differential expression or co-expression analysis (Weighill et al. Gene Targeting in Disease Networks. doi:10.3389/fgene.2021.649942). We have modified the text to provide additional context for the reference:

“Many of these methods share a methodological and philosophical framework that derives from the “No Free Lunch Theorem”—modeling of complex systems can be improved by incorporating domain-specific knowledge [28]—as they optimize around a regulatory network prior and impose biologically motivated soft constraints. Many of these methods also use an overlapping set of standard input data types and provide complementary views of gene regulatory networks.”

- "centralized resource for biological networks" => isn't this more the goal of GRAND or NDEX? Furthermore, this sentence is unclear.

-> We appreciate this comment as this is helping clarify an important question in the manuscript. We wanted to emphasize the fact that developing a robust codebase in GitHUb has enabled the development of an ecosystem of online tools that work together such as GRAND database, Netbooks the Jupyter notebook server, and ZooKeeper the unit test and continuous integration server. Therefore, we rephrased the sentence to make it more clear.

“The netZoo codebase has helped develop an ecosystem of online resources for GRN inference and analysis to both scientists and method developers that includes tools to integrate contributions from the community, to share use cases [29], and to host and visualize networks online [30].”

Results

- The paper lacks description on how the tools were harmonized and adapted to the different languages. This could be a first section, which would also include the description of the community ecosystem that is currently available after the description of the use-cases.

-> Thank you for this great comment, our approach in harmonizing the implementations of existing tools had several aspects:

- Aggregating all methods in one single drop point has allowed us to easily maintain them and reconcile their dependencies. Before this work, combining these methods through their individual packages might require additional steps to reconcile different versions of their dependencies to be able to install and use them.
- We have developed unit tests to guarantee the accuracy of methods and integrated them in a continuous integration server to ensure that contributions from developers are tested thoroughly before being merged in the core codebase.
- Merging code of the same method in different languages in the same resource has allowed us to build unit tests that run across languages. In other words, we used the same ground truth data for R, MATLAB, Python, and C, which has uncovered some inconsistencies between implementations for several edge cases. Ultimately, this helped improve reproducibility and harmonized the methods across platforms.
- We have created interfaces between methods to enable pipeline analyses. We demonstrated this in the paper by using a PANDA-LIONESS-MONSTER combination, which required identifying intersections between methods that could be exploited to take advantage of the synergies between them. This was possible because we standardized I/O formats for networks across methods and made data transformations that would enable compatibility between these tools and fit the underlying statistical assumptions for each method.
- We optimized runtime and memory requirements for these implementations. For example, we created an R wrapper for PANDA Python function because the pure R function was significantly slow despite our attempts to optimize the code, which shows a great example of possible synergies. In a separate work (DOI: [10.1093/nargab/lqac002](https://doi.org/10.1093/nargab/lqac002)), we developed GPU implementations for netZoo that are ~50x faster than CPU.
- Deploying various netZoo packages in R and Python to code repositories (Bioconductor, conda) required optimizing code and package size which further improved usability and code quality.
- Finally, before the development of netZoo, packages for these methods were scattered around the internet and several parallel development branches existed which confused users and impacted reproducibility. netZoo has allowed to merge these branches by resolving any conflict that may have occurred. netZoo also hosts newly developed methods such as DRAGON and that were “natively” developed in netZoo using the same processes described previously to facilitate their use with other methods.

We added the details of these implementations as part of the first results paragraph (netZoo integrates network inference and downstream analyses)., however, we chose to leave the community ecosystem description for the end of the results section because it includes an outlook on where we are anticipating future development of this work.

- The paper lacks description of the different omics datasets that can be used as input for the different methods. Different datasets are also mentioned without description. For instance "gene knockdown" or "fitness". The method section does not provide details neither.

-> As a response to this excellent suggestion, we added a supplementary table (Table S1) for the different omics datasets that can be used with each method. We have also added a detailed description for each dataset we used from CCLE, including the cellular response to CRISPR gene knockout screens also defined as cell fitness screens.

- The method section is not referenced in the Result section. The result and method sections do not provide enough information and details to understand the experiment that have been conducted and the results obtained. The split of the text between method and result sections is sometimes not clear, with methodological details available from both places.

-> We have relocated methodological details that were mentioned in the results to methods section. We have also added references to the methods in the result to better separate these two sections.

As a response to this comment and to previous ones, we have added additional information to better describe the data and the experiments that were conducted in methods, including supplementary method tables (Table S1, S3, and S4). Finally, we expanded the text in result paragraphs that seemed hard to follow with additional details and definitions.

- "assessing significance of differential modules" unclear

-> This sentence was indeed confusing and we restructured it to the following:

“CRANE [26] assesses the significance of differential modules discovered by ALPACA based on a baseline of network ensembles that are simulated while preserving the specific structure and constraints of GRNs. In this regard CRANE provides an efficient tool for hypothesis testing inference on differential community structures in GRNs.”

- "TF targeting" has not been clearly defined. I'm not sure to understand what biological process it corresponds to, and what is the rationale for linking it with outgoing edge weights. Is "TF targeting" and "targeting by the TF" the same process?

-> TF targeting scores are a network metric defined as the weighted outdegree for each TF. We found that this network metric can be useful in summarizing active biological processes in various contexts and we validated them using gene enrichment analysis (Weighill et al, doi.org/10.3389/fgene.2021.649942). Biologically, TF targeting corresponds to the number of targets acquired by that TF in a specific context, which can be defined for example by the total sum of ChIP-Seq peaks in the promoter of target genes.

Taken together, TF targeting loosely reflects “TF activity”. We added these details in the method section and we added examples to support these definitions.

For the second part of the question, “TF targeting” and "targeting by the TF" correspond to the same process, so we rephrased “targeting by TF” to “TF targeting” to avoid any confusion.

-"we assume that if a gene product is repressed by miRNA, then it is likely nonessential": this sounds like a very strong statement, which - a minima - should be backed up by references.

-> We agree with the referee that this statement may be indeed strong and we will attempt to explain our reasoning here and support it with references.

It is well known that miRNA are implicated in gene silencing through mRNA degradation and therefore in reducing the functional effects of the target genes. A strong miRNA repression would be very similar to a complete gene KO using CRISPR, which should be salient through a strong correlation between CRISPR screens and miRNA levels. Small RNAs similar to miRNA (siRNA and shRNA) have been used widely to silence genes for KO/KD experiments.

We added a reference to this sentence in the text and rephrased to “In the first comparisons between miRNA expression and gene knockout, we assume that strong gene silencing by miRNA [9] would share a similar dependency signature with a gene knockout using clustered regularly interspaced short palindromic repeats (CRISPR), as other small RNAs are commonly used in knockdown experiments”

- "with a context+ score of -0.16" this is unclear, it assumes the reader is very familiar with the methods and their outputs.

-> We are sorry for this confusion and have clarified this score in the text. The context+ score has been introduced in 2011 by Garcia and colleagues (PMID: 21909094) and improved upon by Agarwal and colleagues to create the context++ score (PMID: 26267216) as a way to assess the likelihood of interaction between and miRNA and their target mRNA by aggregating information from 14 features including site-type contribution, 3' pairing contribution, local AU contribution, position contribution, TA (target site abundance) contribution and SPS (seed-pairing stability) contribution. This score has been used in TargetScan miRNA database, that we used as an independent validation for miRNA target predictions in our CCLE analysis. Lower context++ scores indicate stronger interaction evidence between miRNA and mRNA and is usually combined with the rank of these predictions. In our case, we gave both the rank and the context++ score for the predicted interaction to give a better idea on the accuracy of our prediction.

We added more details in the sentence to clarify this concept for readers.

- "this suggests that these metabolites are upstream of LDHA" I don't get why.

-> To infer the direction of biochemical reactions in a pathway, one approach would be to correlate metabolite and enzymes levels, there can be 2 different possibilities for each metabolite based on whether it is a product or reactant. By using the canonical glycolysis pathway as a template, we can infer the direction of each reaction using the following fundamental relation

We added this schematic in figure S2. Here, we treated the case of positive correlations between enzyme and metabolite levels, but its dual case for negative correlation gives the reverse direction.

Therefore, to extrapolate from this example to our case to find the direction of the TCA cycle reactions, we correlated metabolite levels with LDHA, a key enzyme that acts as a bottleneck in the pathway. LDHA directs energy production either to TCA cycle or fermentation (Warburg effect in cancer cells). We have mentioned that in the discussion that these results are only suggestive and need further validation, but it is a an example of combining network theory and multiomic data to identify a common hallmark of cancer.

- "this effect could be dominant in CCLE, since we did not filter ..." I don't get why.

-> Our reasoning to infer the dominance of this effect was because we used metabolite and enzyme levels in all cell lines from all lineages to build the multi-omic partial correlation map and identify pan-cancer effects common to all cell lines, without filtering for a specific phenotype. The Warburg effect suggested in this analysis seems to be dominant since this is based on a correlation analysis and therefore detects effects that are present in a majority of cell lines. We amended the sentence in the text to make it a more clear.

- Are the ANOVA and ElasticNet approaches presented here implemented in netZoo?

-> No, ANOVA and ElasticNet are popular algorithms for linear regression.

ANOVA is a generic method for linear regression on categorical variables. In our case, these categorical variables are methylation status (1=hyper, 0=hypo), copy number variation status (1=hyper, 0=hypo).

ElasticNet is a penalized linear regression that allows the selection of variables in highly correlated datasets such as genomic data.

We used both these methods for downstream analysis. We appreciate the referee’s comment pointing to this lack of clarity and we added citations and references to their original implementations to indicate that they are not part of netZoo..

-"we systematically overlaid networks based on our understanding of the cascade of regulatory processes": how? Manually?

-> We have manually overlaid DRAGON network being guided by the central dogma of biology and common understanding of the flow of information between omic layers, e.g., transcriptomics->proteomics->metabolomics. We did this to better annotate the DRAGON multiomic graph which is undirected; however we added directions using prior biological knowledge. We have rephrased this sentence adding more details to reflect our approach. We’ve also added a reference to the methods section and a supplementary figure (S3) where we described our approach in detail.

Conclusion

- "multi-omics gene regulatory and partial correlation networks": the semantic used here is different from the one used in the abstract and introduction, not clear what method/approach it refers to.

-> In this sentence, We are referring to the DRAGON multiomic CCLE network that we presented as a use case in figure 4 and as an interactive map here <https://grand.networkmedicine.org/cclemap/>. This network is a multiomic network based on partial correlation. We have clarified this sentence in the conclusion paragraph and resolved any inconsistency with the abstract and introduction.

- "in line with recent similar effort": the conclusion does not seem the be the correct place to mention existing effort and state of the art, that are in addition lacking in the background section

-> Thank you for this great suggestion, we relocated this sentence to the background section. The background now mentions the state of the art as it relates to gene regulatory network inference, and as it relates to recent efforts in combining software tools in unified packages.

- "sharing of use cases", "continued development and maintenance", "community participation", these have not been described, to the best of my knowledge.

-> These concepts have been described in the result/discussion section “Creating a community ecosystem for collaborative software development”.

We have clarified this sentence by expanding the discussion to the specific tools we refer to for sharing use cases (Netbooks), continuous development (ZooKeeper), ad community participation (GitHub discussion and collaborative development model).

- "hosting networks" isn't this more the goal of GRAND than of netZoo?

-> This is indeed correct. We define netZoo here as the codebase of methods that we have aggregated in GitHub and improved upon. We also refer to the “netZoo ecosystem” (Figure 5) as the set of online resources that is comprised of the core netZoo GitHub codebase, a continuous integration system and unit tests called ZooKeeper, GRAND database, and Netbooks which is the Jupyter Notebook server.

Our ability to unify the codebase in a single drop point has allowed us to build this ecosystem of tools, which wouldn’t be possible without these efforts, which ultimately improved robustness and reproducibility of our methods.

For each of our current investigations, we now deposit the code on GitHub, upload the networks to GRAND, and upload the code to reproduce statistical analysis and figures on Netbooks. Therefore readers have instantly access to an unparalleled level of reproducibility for various types of data and code that they can use for their own work, without having to redo the analyses.

For the future, we envision to add more components to this ecosystem of online tools and to build connections between them. For example, we foresee a tool that, upon updating the core codebase, would automatically regenerate networks in GRAND and reruns analyses in Netbooks and version-controls them as new releases.

We clarified this point further in the conclusion of the manuscript.

Methods

- method section is not presented in the same order than the results

-> The method section was presented in the same order as the results, we are sorry for this confusion that we believe might have happened because we did not clearly state in the result headings that the melanoma case study was based on CCLE data, so we added this mention in the results.

The use case application in this paper was to apply the combined capabilities of netZoo methods to analyze CCLE data because it presents some challenges that biologists might have to face in analyzing data in an increasingly multi-omic environment.

We presented three sub-aims in CCLE data analysis, first starting with cancer-specific analysis in melanoma, second by conducting a pan-cancer analysis across all cancer cell lines, and finally by building a multi-omic aggregate network.

The methods section has four sections starting first by describing the CCLE data, then describing the data for the first melanoma use case, then the second pan-cancer analysis, and finally the multiomic CCLE network. As such it does perfectly mirror the organization of results:

- Methods section “netZoo applications using the Cancer Cell Line Encyclopedia” has CCLE data description and parameters for network generation and other definitions that were used throughout the manuscript to avoid repeating them in other sections of the methods.
- Results: Estimating TF targeting in melanoma CCLE cell lines -> Methods: TF targeting analysis
- Results: CCLE pan-cancer analysis reveals meaningful regulatory interactions -> Methods: Computing CCLE multi-omic associations
- Results: An integrated CCLE multi-omic network portal -> Methods: CCLE pan-cancer map
- For result headings (“Creating a community ecosystem for collaborative software development” and “netZoo integrates network inference and downstream analyses”), although they tend to be more methodological than a classical results section, we believe that since this a “Software” paper, a stronger emphasis should be put on the software package and its development as an output and a result of this work.

-"running FIMO": is FIMO also available in netZoo?

-> FIMO is not available in netZoo. FIMO is a software tool developed by the Noble research group at the University of Washington (we have added the corresponding citation next to it in the text). We used FIMO to generate input data for netZoo methods such as PANDA to detect the presence of TF footprint (or DNA binding motif) in the promoter region of a target gene. FIMO is the tool that runs these scans using position weight matrices (PWM) database such as CIS-BP and HOCOMOCO.

We may use various tools developed by the community to support netZoo analysis. This can take palace either for input data processing for netZoo tools such as tools for gene expression analysis, batch correction, and data normalization. We also use tools for downstream analysis after data generation which include igraph for network analysis, various types of regressions such as ElasticNet or LIMMA for differential analysis and cox regression for survival analysis.

-"gene-gene correlations network based on expression", more details on how the correlations are computed, from what type of data, would be pertinent.

-> To clarify this sentence, emphasizing the methodological details, we rephrased it to:

“In addition, we used as inputs a TF PPI network derived from the STRING database [2] (using the aggregate score for human interactions only and scaling them between 0 and 1) that we restricted to a list of 1,603 TFs as defined by Lambert and colleagues [5], and a gene coexpression network between 19,177 protein-coding genes across 1,376 cells. The latter network is a Pearson correlation network based on RNA-Seq data as preprocessed in CCLE by adding a pseudo-count of 1 to TPM gene expression data and applying Log2 transformation.”

-"using the mode parameter to intersection which take the intersection" not clear

-> Thank you for pointing this out, the sentence was cut during the editing process, we rephrased it to

“The resulting PANDA network includes regulatory associations between 1,132 TFs and 18,560 genes because we set the ‘mode’ parameter in PANDA to ‘intersection’ which takes the intersecting TFs and genes between the three input networks.”

- ANOVA model: why discretizing the data before doing the ANOVA, and not using the raw data?

-> CCLE has indeed provided quantitative data about copy number variation (CNV) and promoter methylation and is therefore amenable to quantitative analysis using the raw continuous data. In our use case, we chose to do a semi-quantitative analysis by discretizing the CNV data and the methylation data and attempt in a first approach to recover known hallmarks of melanoma using ANOVA (that requires categorical data as explanatory variables) to validate the accuracy of our methods. ANOVA is interesting in this case because it allows to recover associations between explanatory variables and a response, compute their significance, and estimate their effect size. With ANOVA, we can make statements of the type “Hypermethylation of promoter of gene A will increase targeting of TF B by X”, whereas if we did a quantitative analysis using linear regression for example, we would have had statement of the type “Increasing methylation of promoter of gene A by Y will increase targeting of TF B by X”, which doesn’t change the results for us because we are mining for associations at this stage.

We rephrased the sentence as follows:

“Since we were mostly interested in finding strong associations and prominent regulatory hallmarks of melanoma, we discretized the input data by considering a gene to be amplified if it had more than three copies and to be deleted if both copies are lost.”

However, in the following step, we conducted a quantitative analysis using Elastic Net regression of drug viability profiles on TF targeting scores (Figure 2B) because we estimated that this use case would require more precision.

- "positive instance for methylated promoter/amplified genes and negative instances for nonmethylated promoters/nonamplified genes" this is counter intuitive to me as methylation inhibits genes expression, contrarily to gene amplification. What is the rationale for pairing opposite biological signals?

-> The referee’s reasoning is indeed correct, however we did not combine these data together in a single model. Our approach was to build an ANOVA model for each modality on its own. Therefore, we had 4 ANOVA models (hypermethylation, hypomethylation, amplification, and deletion) that we fit separately.

To clarify this point, we modified this section as follows:

“For example, in modeling promoter hypermethylation, we chose positive instances to represent hypermethylated promoters and negative instances for nonmethylated promoters along with an additional factor correcting for the cell lineage. Similarly, for copy number variation analysis, we chose positive instance to represent amplified genes and negative instances for nonamplified genes while correcting for cell lineage. We only computed the associations if they had at least three positive instances of the explanatory variable e.g., promoter hypomethylation in at least three cell lines.”

- Data used from CCLE are poorly described.

-> As a response to this excellent suggestion, we have added a detailed description of each dataset taken from CCLE in the first paragraph of the methods section.

We also added Table S3 which includes CCLE references and the URLs to download the data.

Figure 1

- arrows are not clear, for instance it seems that gene co-expression is used only by PANDA and not by OTTER.

-> We separated the arrows for each method. To avoid confusion, we annotated the arrows by numbers to avoid crossing them.

- description of omics data used by the different methods could be improved.

-> As a response to this comment, we added in the figure a second line in the description of each method to identify the data used between parentheses. In the figure caption, we described each one of the omics data in more detail.

- why are some tools overlapping (eg PANDA and OTTER) and not others?

-> There is an overlap when tools use exactly the same input data. We clarified this in the figure caption.

- is the output of SAMBAR linked to MONSTER?

-> These outputs are not linked together, however it can be understood in this way because there is no space between SAMBAR and MONSTER. We separated them in the figure to avoid any confusion.

- ALPACA/CRANE why two methods for one goal?

-> There is a slight difference between them. CRANE computes the significance of network modules identified by ALPACA. We resolved this point in the updated figure.

- Some methods are described in the caption but not in the figure.

-> This is a great point, DRAGON was indeed missing in the figure and so we added it in the updated version.

- There are some discrepancies between the caption and the figure, in the description of the methods. For instance, MONSTER "estimates a transition matrix between two networks" or "estimates network transition drivers". This lead to unnecessary complexities.

-> We resolved these inconsistencies between the caption and the figure. We did this for MONSTER, YARN, ALPACA, and CRANE to match their figure and text description..

Figure 4B and C, edge colors are not defined.

-> As a response to this comment, we defined edge colors in the figure caption. Positive partial correlation are colored in green and negative partial correlation are colored in red.

**Reviewer #3:**

Inferring gene regulatory networks from collections of omics profile is a critical component in studying complex molecular phenomena underlying health and disease. Over the years, the Quackenbush has developed an incredible array of methods and software tools to reconstruct, compare, and contrast context-specific such networks. In this manuscript, they describe netZoo, a unified software framework that harmonizes the implementations of all these tools under one roof, enabling researchers to apply them in an integrative manner. With implementations in multiple programming languages, tools to ensure code consistency and robustness, and features for community support, netZoo is likely to have major impact.

At this point, though the achievements of this effort can be inferred, the manuscript is in serious need of some key details and descriptions.

Major comments/questions

=================

1. What did harmonizing the previous implementations of these tools entail despite them sharing common methodologies and computational cores? What were the challenges in this harmonization process in terms of the underlying analytical methods, data structures, numerical routines, memory/space constraints, etc.?

-> Thank you for this great comment, our approach in harmonizing the implementations of existing tools had several aspects:

- Aggregating all methods in one single drop point has allowed us to easily maintain them and reconcile their dependencies. Before this work, combining these methods through their individual packages might require additional steps to reconcile different versions of their dependencies to be able to install and use them.
- We have developed unit tests to guarantee the accuracy of methods and integrated them in a continuous integration server to ensure that contributions from developers are tested thoroughly before being merged in the core codebase.
- Merging code of the same method in different languages in the same resource has allowed us to build unit tests that run across languages. In other words, we used the same ground truth data for R, MATLAB, Python, and C, which has uncovered some inconsistencies between implementations for several edge cases. Ultimately, this helped improve reproducibility and harmonized the methods across platforms.
- We have created interfaces between methods to enable pipeline analyses. We demonstrated this in the paper by using a PANDA-LIONESS-MONSTER combination, which required identifying intersections between methods that could be exploited to take advantage of the synergies between them. This was possible because we standardized I/O formats for networks across methods and made data transformations that would enable compatibility between these tools and fit the underlying statistical assumptions for each method.
- We optimized runtime and memory requirements for these implementations. For example, we created an R wrapper for PANDA Python function because the pure R function was significantly slow despite our attempts to optimize the code, which shows a great example of possible synergies. In a separate work (DOI: [10.1093/nargab/lqac002](https://doi.org/10.1093/nargab/lqac002)), we developed GPU implementations for netZoo that are ~50x faster than CPU.
- Deploying various netZoo packages in R and Python to code repositories (Bioconductor, conda) required optimizing code and package size which further improved usability and code quality.
- Finally, before the development of netZoo, packages for these methods were scattered around the internet and several parallel development branches existed which confused users and impacted reproducibility. netZoo has allowed to merge these branches by resolving any conflict that may have occurred. netZoo also hosts newly developed methods such as DRAGON and that were “natively” developed in netZoo using the same processes described previously to facilitate their use with other methods.

We would like to clarify a point in the referee’s comment that sharing “common methodology” reflects sharing “a similar modeling paradigm” rather than sharing “similar code.” This paradigm suggests that using knowledge as a prior to seed network inference can yield more accurate results.

We added the details of these implementations as part of the first results paragraph (netZoo integrates network inference and downstream analyses).

2. The authors lay out a detailed CCLE analysis. But, this whole description is not serving the main point this manuscript is hoping to make, which is: how did this extensive analysis become possible and/or easier with netZoo and why wasn't is possible to do exactly the same analysis just using the individual tools as they were available from their previous implementations? In the context of this manuscript, the authors need to rewrite to use these cool analyses as a backdrop to talk about the challenges in using existing implementations and what specific things they developed in netZoo that enabled them to overcome these challenges.

-> netZoo includes two types of methods: A) those that were published previously as individual packages and were aggregated later in the netZoo, B) tools that were uniquely published in netZoo. The CCLE analysis includes methods from both A and B categories. So we organized our response in the

I. Building interfaces between existing methods.

For the first category, these tools have been previously used individually (PANDA, LIONESS, MONSTER) and have been used in combination such as PANDA+LIONESS for single-sample network reconstruction. The success of combining these two methods was our motivation in creating netZoo because we were interested in finding new synergies between methods that would ultimately give new biological insights. And so in the use case presented in this paper, we added a MONSTER network transition analysis on top of the PANDA-LIONESS inferred networks. This wouldn’t have been possible previously because we developed an interface within netZoo that links MONSTER with PANDA-LIONESS. The original implementation of MONSTER had an initial step that generated two gene regulatory networks using gene expression data and a second step that estimates transition between them. Reimplementing MONSTER in netZoo allowed the creation of a natural way to link the transition analysis to PANDA-LIONESS networks.

II. Improving reproducibility of existing methods

netZoo made this analysis easier to execute by aggregating all methods in one package that combines all the dependencies, which is easier for installing and running code. Also by developing an extended set of unit tests for each method, we were able to detect and fix inconsistencies in edge cases, particularly by reconciling implementations in different programming languages. netZoo implementations are optimized for computational resources (runtime and memory) including GPU implementations, and they are more accurate and stable than older versions.

III. Novel applications with new methods

netZoo includes a set of novel tools (DRAGON, EGRET, OTTER) that were not published elsewhere. Developing these tools took advantage of the infrastructure already built (unit tests, integration with other methods) to provide users with a better programming experience. In the CCLE use case, we used DRAGON as an example to model complex multiomic data.

IV. Creating a community of users and developers

Although this is not a direct outcome on the CCLE analysis, aggregating methods in unique place through netZoo has helped create an “agora” of users and developers. The net effect of this was the faster detection of errors and bugs that may occur, new ideas for method integration, which results in better code quality.

V. Taking advantage of the uniqueness of CCLE data

We chose CCLE data as our use case because despite having information about more than 7 omics across more than a thousand cell line, gene regulation was not characterized. This shows that computational modeling can be an alternative for the lack of large-scale experimental techniques to measure the activity of regulatory elements.

CCLE data is the perfect example to show that netZoo is uniquely positioned to model complex multiomic data. In particular, CCLE has one single gene expression sample for each cell line, making it impossible for aggregate network methods (coexpression, ARACNE, WGCNA) to model them. However, LIONESS can model networks using a single gene expression sample using population data as a background. Finally, modeling multiomic data across 7 omics is a very challenging task because each omic has a unique data structure. These differences in distributions have to be accounted for, in order to detect biological signals and find meaningful associations in the high-dimensional data. DRAGON was designed for this specific purpose by using a technique called covariance shrinkage to account for different data structures across omics when computing partial correlations between variables.

We summarized these points in the results section “netZoo integrates network inference and downstream analyses” and “Estimating TF targeting in melanoma CCLE cell lines”.

3. The development team has done a commendable job making many tools within netZoo available in multiple programming languages. However, why did they end up with different subsets of tools being implemented in R, Python, MATLAB, and C? The question is not to ask for all tools in all languages. Instead, the authors need to discuss this in the manuscript: how and why did the authors make these choices of tools for different languages.

-> As a response to this excellent comment and to feedback by other referees, we chose one language (R) that would have all implementations of all thirteen methods and therefore we additionally implemented SPIDER, PUMA, and DRAGON in R and we hope to have broader representation of methods in Python in the near future.

Although using various languages does not impact the combined use of these methods because pipeline tools such as Nextflow allow to combine various programming languages, we chose to expand implementations in this revision and we are planning to release our Nextflow pipeline in the future.

The reason we had various implementations was driven by preferences of developers in our extended community. The first implementations were done in C, and then for ease-of-use, they were translated to scripting languages, first to MATLAB for its fast matrix multiplication capabilities, and then to R for the statistical packages that enable easier downstream analyses and plotting functions. We have noticed that R was significantly slower than Python for matrix multiplication despite our attempts to optimize code and linear algebra libraries and so Python seemed to offer a good compromise for an open-source platform and with excellent performance.

The netZoo community spans several academic groups and a large number of users, therefore, we expect such variety in preferences, although R and Python are the most used netZoo packages.

We mentioned this point in the last result section “Creating a community ecosystem for collaborative software development”.

Minor comments/questions

=================

1. The various tools — from PANDA to MONSTER — are described to different extents more than once in the manuscript. This is unnecessary. In fact, a single detailed description of each tool can be put together in one place and provided as part of the supplement, leaving just a high-level outline in the main text.

-> As suggested by the referee, we removed the second occurrence of the method description which was mentioned in the results section, and we expanded on the descriptions in the background section.

Finally, we added more detailed descriptions in the supplementary file and referred the reader to them in the main text.

2. Page 9, end of the first paragraph under "Modeling TF targeting associations in melanoma": … if it had a z-score of less than *negative* three.

-> We fixed this typo as suggested by the referee.

**2^nd^ round**

**Reviewer 2**

I do not have additional comments, I congratulate the authors for this excellent revision.
